# PRMT5 inhibition triggers functional ATM deficiency and sensitizes pancreatic cancer to CHK1 blockade

**DOI:** 10.3389/fcell.2026.1748541

**Published:** 2026-03-11

**Authors:** Madeline Dzikowski, Gareth Pollin, Veda Gunia, Shawna Butler, Byambasuren Enkhtuul, Jennifer Gavina Chavez, Michael T. Zimmermann, Angela Mathison, Raul Urrutia, Gwen Lomberk

**Affiliations:** 1 Department of Pharmacology and Toxicology, Medical College of Wisconsin, Milwaukee, WI, United States; 2 Linda T. and John A. Mellowes Center for Genomic Sciences and Precision Medicine, Medical College of Wisconsin, Milwaukee, WI, United States; 3 Department of Surgery, Division of Research, Medical College of Wisconsin, Milwaukee, WI, United States; 4 Data Science Institute, Medical College of Wisconsin, Milwaukee, WI, United States; 5 Departments of Biochemistry, Biophysics, and Physiology, Medical College of Wisconsin, Milwaukee, WI, United States

**Keywords:** ATM, combination (combined) therapy, DNA damage response (DDR), epigenetic therapeutics, PDAC (pancreatic ductal adenocarcinoma), PRMT5

## Abstract

**Introduction:**

PRMT5 inhibitors are under clinical investigation for pancreatic ductal adenocarcinoma (PDAC), but strategies to maximize their therapeutic efficacy remain undefined. Here, we sought to determine whether pharmacologic inhibition of PRMT5 creates therapeutically exploitable vulnerabilities in PDAC.

**Methods:**

We employed immunofluorescence, western blotting, and comet assays for molecular analyses, Incucyte live-cell imaging and clonogenic assays for in vitro growth assessment, RNA-seq for transcriptomic profiling followed by RT-qPCR array validation of selected targets, and xenograft models with immunohistochemistry to evaluate in vivo effects.

**Results and discussion:**

We report that pharmacologic inhibition of PRMT5 markedly reduces ATM levels across multiple PDAC cell lines using different PRMT5 inhibitors, resulting in a functional ATM-deficient state. This reduction in ATM rewires DNA damage response signaling, increasing PDAC cell reliance on the ATR-CHK1 pathway for survival. Consequently, we tested combined PRMT5 and CHK1 inhibition and found synergistic suppression of PDAC growth, accompanied by enhanced Caspase 3/7 activation, Annexin V staining, and DNA damage accumulation. Congruently, RNA-seq demonstrated downregulation of cell-cycle and DNA repair genes along with upregulation of cell death pathways, providing mechanistic insight into the cooperative effect. Moreover, in subcutaneous xenografts, the combination substantially reduced tumor volume, prolonged median survival, and did not adversely affect body weight. Treated tumors showed reduced ATM and Ki67 with elevated γH2A.X. Together, these findings identify PRMT5 inhibition as a trigger of reduced ATM function that exposes a therapeutically actionable vulnerability to CHK1 inhibition, offering a rational, mechanistically-based combination strategy for this dismal disease.

## 1 Introduction

Pancreatic ductal adenocarcinoma (PDAC) remains among the most lethal cancers, ranking as the third leading cause of cancer-related deaths in the United States, with a 5-year survival rate of only 13% (Duggan et al., 2016). Late-stage diagnosis, limited surgical options, high recurrence rates, and aggressive tumor biology contribute to its poor prognosis, highlighting the urgent need for innovative treatment strategies. Although recent advances in precision oncology have enabled targeted treatment for select PDAC subgroups with actionable mutations (Lomberk et al., 2019; Li et al., 2024; Mouawad et al., 2024), most patients still lack effective options. Parallel efforts to exploit epigenomic vulnerabilities are emerging to complement other approaches, particularly when combined with agents targeting genetic or signaling dependencies, thereby broadening the treatment landscape for this recalcitrant disease.

Our laboratory has focused on leveraging cell-cycle specific chromatin regulation to uncover unique vulnerabilities in PDAC. Chromatin modifiers often exert distinct, stage-dependent functions that can be exploited to reveal functional dependencies. For example, we previously showed that Aurora kinase inhibition exposes H3K9me3 at centromeric chromatin. This exposure creates a vulnerability that can be targeted by histone methyltransferase inhibitors, driving PDAC cells into mitotic catastrophe (Mathison et al., 2017). Similarly, inhibiting the H3K9 methyltransferase G9a/EHMT2 impairs replication stress tolerance, thereby increasing sensitivity to CHK1 inhibition and synergistically suppressing PDAC tumor growth (Urrutia et al., 2020). Together, these studies illustrate the therapeutic potential of mechanistically informed combinations that target both epigenetic and checkpoint regulation. Such combinations can enhance antitumor efficacy by coordinating these pathways and can also reveal functional interactions that are not apparent when each pathway is perturbed in isolation.

Building on this rationale, we investigated the type II arginine methyltransferase PRMT5, an enzyme that symmetrically di-methylates histones and numerous non-histone proteins (Kim and Ronai, 2020). PRMT5 is frequently upregulated in PDAC, and its elevated expression correlates with reduced disease-free and overall survival in patients (Qin et al., 2019; Wei et al., 2020; Orben et al., 2022). Due to its central role in regulating chromatin organization, RNA splicing, ribosome biogenesis, and cell-cycle progression, PRMT5 has emerged as a compelling therapeutic target, with several inhibitors advancing through clinical testing (Scoumanne et al., 2009; Ren et al., 2010; Jahan and Davie, 2015; Fong et al., 2019; Watts et al., 2019; Jin et al., 2025). Previous studies have shown that PRMT5 supports the DNA damage response (DDR) by promoting transcription of key DDR genes, including RAD51, BRCA1, and BRCA2 (Owens et al., 2020), and that its loss compromises DNA repair capacity (Hamard et al., 2018). However, how PRMT5 inhibition specifically reshapes DDR signaling in PDAC remains poorly defined. In this study, we show that pharmacologic inhibition of PRMT5 induces a state resembling ATM haploinsufficiency. This is particularly relevant in PDAC because partial loss or functional impairment of the DDR kinase ATM occurs in a distinct subset of tumors and is linked to genomic instability and adverse clinical features (Armstrong et al., 2019; Gower et al., 2021; Hannan et al., 2021). When ATM signaling is compromised, DDR control shifts toward ATR-CHK1 signaling, creating a potential therapeutic vulnerability that can be exploited. These ATM-dependent alterations in DDR capacity likely contribute to the heterogeneous responses to DNA-damaging therapies observed in patients with PDAC. Accordingly, we tested whether PRMT5 inhibition would sensitize PDAC cells to CHK1 blockade. We found that combined PRMT5 and CHK1 inhibition reduced cell growth, increased apoptosis and DNA damage, altered transcriptional programs governing cell-cycle and DNA repair, and suppressed tumor progression with prolonged survival *in vivo*. Collectively, our study positions PRMT5-CHK1 co-inhibition as a promising therapeutic strategy that expands epigenomic targeting approaches to overcome compensatory DDR dependencies in PDAC.

## 2 Materials and methods

### 2.1 Cell lines and reagents

L3.6 PL were obtained from MD Anderson (Bruns et al., 1999; Nakamura et al., 2007). hTERT-HPNE E6/E7/K-RasG12D/st (CRL-4039), MiaPaCa2 (CRL-1420), BxPC3 (CRL-1687), PANC1 (CRL-1469), Panc10.05 (CRL-2547), and Panc03.27 (CRL-2549) cells were obtained through ATCC. All cells were cultured according to their manufacturer’s specifications. EPZ015938 (Cat #: S8664), an analog of EPZ015666, was obtained from Selleck Chemicals, JNJ-64619178 (Cat #: HY-101564) and MRTX9768 (Cat #: HY-138684) were obtained from MedChemExpress, and prexasertib (Cat #: B1088) was obtained from APExBIO. Drugs were reconstituted in DMSO. Antibody information for all relevant experiments is provided in Supplementary Table S1.

### 2.2 Live-cell growth and drug synergy calculations, live-cell fluorescent apoptosis, and clonogenic survival assays

For the live-cell growth assays, cells were plated in a 96-well plate and treated with EPZ015938 and prexasertib at indicated concentrations for up to 7 days. Images were acquired every 6 h using the IncuCyte SX5 Live-Cell Analysis System, and cell viability was inferred from confluence measurements. Viability was compared among the groups using one-way ANOVA with Tukey’s multiple-comparison *post hoc* analysis. Data are shown as mean ± SEM, and p < 0.05 was considered statistically significant. Drug interaction synergy was quantified using multiple synergy reference models (Bliss, Loewe, and Highest Single Agent/HSA) with the SynergyFinder3.0 web application (Ianevski et al., 2022). For live-cell fluorescent apoptosis assays, Caspase 3/7 green fluorescent dye (Sartorius, 4440), Annexin V red fluorescent dye (Sartorius, 4641), and CytoTox dead cell NIR fluorescent dye (Sartorius, 3846) were prepared in culture media according to manufacturer’s instructions. Cells were seeded in 96-well plates, treated with drug-containing media supplemented with the multiplexed dyes, and imaged every 3 h for 4 days using the IncuCyte SX5. Relative fluorescent area was compared among the groups using one-way ANOVA with Tukey’s multiple-comparison *post hoc* analysis. Data are shown as mean ± SEM, and p < 0.05 was considered statistically significant. For clonogenic assays, cells were seeded in 6-well plates and exposed to drug treatments, with media and treatments replaced on days 4 and 7. On day 11, cells were fixed with acetic acid:methanol (1:7) containing 0.5% crystal violet, and colonies were imaged and quantified using the ColonyArea ImageJ plug-in (Guzmán et al., 2014). Colony area was compared among the groups using one-way ANOVA with Tukey’s multiple-comparison *post hoc* analysis. Data are shown as mean ± SEM, and p < 0.05 was considered statistically significant.

### 2.3 Immunofluorescence and confocal microscopy

Cells were plated on L-poly-lysine-coated coverslips and treated with drug-containing media for up to 5 days. Cells were then fixed with 4% paraformaldehyde in PBS for 30 min, permeabilized with 0.2% Triton X-100 in PBS for 20 min and blocked with 1% BSA in PBS for 1 h. Coverslips were incubated with primary and secondary antibodies according to the manufacturer’s recommendations, counterstained with DAPI (1:5000) and mounted onto microscope slides using ProLong Gold Antifade Mountant (Thermo Fisher Scientific, P36930). Slides were imaged using a Zeiss confocal microscope at ×63 magnification, and images were quantified using ImageJ. A minimum of 200 cells per condition across 3 biological replicates were quantified for mean intensity fluorescence calculations. Treatment groups were analyzed using Kolmgorov-Smirnov t-test. Data are shown as mean ± SEM, and p < 0.05 was considered statistically significant.

### 2.4 Western blot analysis

Western blots were performed as described previously (Urrutia et al., 2020). Membranes were blocked with 5% BSA in 0.1% TBS-Tween 20 and incubated overnight at 4 °C with primary antibodies (Supplementary Table S1), followed by anti-mouse or anti-rabbit secondary antibodies conjugated to HRP or fluorophores, as appropriate. HRP blots were developed using SuperSignal West Pico PLUS Chemiluminescent Substrate (Thermo Fisher Scientific, 34580). All blots were imaged on a Bio-Rad ChemiDoc MP Imaging system with Bio-Rad ImageLab software.

### 2.5 BrdU proliferation assays

Cells grown on coverslips were treated for 72 h, then pulsed with 100 μmol/L BrdU (Sigma-Aldrich) for 1 h. Cells were fixed with 3.7% formaldehyde for 15 min and permeabilized with 0.5% Triton X-100 in PBS for 20 min. DNA was denatured sequentially with 2N HCl for 1 h at 37 °C, neutralized with pH 7.4 phosphate/citric acid buffer, and washed three times with PBS. Cells were blocked with 1% BSA in PBS-Tween 0.1% for 1 h, incubated with anti-BrdU overnight at 4 °C, and then with Alexa Fluor 546-conjugated anti-mouse secondary antibody for 1 h at room temperature. Coverslips were counterstained with DAPI and mounted using ProLong Gold Antifade (Thermo Fisher Scientific, P36930). A minimum of 3500 cells per condition across 4 replicates were manually counted for determining percentage of nuclei positive for BrdU. Change in positive nuclei was compared among the groups using Kruskal–Wallis H-test with Dunn’s multiple-comparison *post hoc* analysis. Data are shown as mean ± SEM, and p < 0.05 was considered statistically significant.

### 2.6 Comet assays

DNA double-strand breaks were assessed using the Trevigen CometAssay Kit under neutral conditions. Single-cell suspensions (1 x 10^5^ cells/mL) were prepared in PBS, mixed with agarose at a 1:10 ratio, and 50 uL/well was pipetted onto slides. Slides were incubated in lysis solution for 1 h and washed in 1X TBE buffer for 15 min. Electrophoresis was conducted under neutral conditions in 1X TBE buffer at 21 V for 40 min. Slides were dried, stained with SYBR Gold I Nucleic Acid stain (Invitrogen, S7563) for 30 min, and imaged by Keyence epifluorescence microscopy using a 488-nm filter. DNA damage was quantified manually with QuPath (v0.5.1) (Bankhead et al., 2017) to calculate mean tail moment, with at least 100 cells analyzed per biological replicate for each treatment condition. Treatment groups were analyzed using log-transformed one-way ANOVA with Tukey’s multiple-comparison *post hoc* analysis. Data are shown as mean ± SEM, and p < 0.05 was considered statistically significant.

### 2.7 Subcutaneous xenografts

Animal experiments were approved by the Medical College of Wisconsin Institutional Animal Care and Use Committee (AUA00006130), following the guidelines in the Guide for Care and Use of Laboratory animals (NIH, Bethesda, MD). Mice were housed in groups of up to five per cage under standard housing conditions (14/10-h light/dark cycle, controlled temperature and humidity), with *ad libitum* access to standard rodent chow and water. Right flanks of athymic nude NU/J mice (*Foxn1* ^*nu*^; 6–8-week old females; The Jackson Laboratory) were injected with 1x10^6^ L3.6 PL cells in 1:1 PBS/Matrigel (Corning). When tumors reached an average volume of 100 mm^3^, mice were randomized by Studylog® (Studylog Systems, Inc.) into groups (n = 5/group) based on tumor volume and body weight. The study was performed twice independently, resulting in 10 animals per treatment group. This sample size provided ∼80% power to detect a 20%–30% reduction in tumor growth or a hazard ratio of roughly 0.55 for survival (α = 0.05). Animals were given EPZ015938 or its vehicle (50 mg/kg in 1:10 DMSO:0.5% methylcellulose) daily via oral gavage, prexasertib or vehicle (2 mg/kg in 20% Captisol) subcutaneously for 3 days followed by 4 days off, with this 7-day cycle repeated for the duration of the experiment, or a combination of the two treatments. Caliper tumor and body weight measurements were made twice weekly. Tumor volumes were calculated using the formula (length x width^2^)/2, where length is the longest dimension and width is the measurement perpendicular to the length. Tumor growth (fold-change in volume at day 17) was compared among the four groups (control, prexasertib, EPZ015938, and combination) using a one-way ANOVA. When the overall ANOVA was significant, Tukey’s multiple-comparison test was used for comparisons between treatment groups. Data are shown as mean ± SEM, and p < 0.05 was considered statistically significant. All randomized animals were included in the analysis with no exclusions. Survival was defined as time from treatment initiation until animals reached humane endpoint criteria (tumor ≥1,500 mm^3^, ulceration, or distress) and was analyzed using Kaplan-Meier curves with log-rank tests for group comparisons. Tumor tissues were formalin-fixed and paraffin-embedded.

### 2.8 Immunohistochemistry

Tumor tissues were processed and stained for hematoxylin/eosin, Masson’s trichrome, Ki67, P-S139-H2AX, and ATM by the Children’s Hospital of Wisconsin Research Institute Histology Core. Slides were imaged using a Hamamatsu microscope, and the full tissue slice was analyzed with QuPath (v0.5.1) (Bankhead et al., 2017) or the ImageJ Color Deconvolution2 plug-in (Ruifrok and Johnston, 2001). Using automated object classification, cells were labelled either as tumor or necrosis, and then using automated cell detection, the percentage of cells positive or negative (based on the default nuclear optical density threshold of 0.2) for DAB staining was calculated. Treatment groups were analyzed using Brown-Forsythe and Welch one-way ANOVA with Dunnett’s multiple-comparison *post hoc* analysis. Data are shown as mean ± SEM, and p < 0.05 was considered statistically significant.

### 2.9 RNA-seq and RT-qPCR

Total RNA from L3.6 PL treated for 5 days with indicated EPZ and prexasertib concentrations was isolated with libraries prepared (TruSeq mRNA Stranded kit, Illumina) and sequenced in the Medical College of Wisconsin Mellowes Center Genomics Core (RRID:SCR 022926) using the NovaSeq 6000 platform (200 cycles, 2x100 bp paired-end reads). Reads were aligned to the human reference transcriptome (Gencode v43, GRCh38), with a minimum of 24 million mapped read pairs per sample. Sequencing reads were processed through the Mellowes Center workflow including MapRseq3 (Kalari et al., 2014) and differential expression calculated by EdgeR (McCarthy et al., 2012), with differentially expressed genes (DEGs) defined as those with a false discovery rate (FDR) < 0.05 and an absolute fold change (FC) ≥ 2 called relative to vehicle control. Pathway analysis of DEGs was performed in Partek using the Molecular Signatures Database (MSigDB) cancer hallmark gene set (Liberzon et al., 2015), as well as KEGG (Kanehisa et al., 2022; 2025), Gene Ontology (Ashburner et al., 2000; Consortium et al., 2023), and Reactome (Milacic et al., 2024), databases. Pathways with q-values <0.05 were considered significantly enriched. For RT2 Profiler RT-qPCR arrays (Qiagen, 330231/PAHS-029Z), cDNA was generated using RT2 First Strand cDNA Synthesis kit (Qiagen, 330401), and RT-PCR was performed according to the manufacturer’s instructions with SYBR Green qPCR Master Mix (Qiagen, 330504) on the CFX96 Real Time System (Bio-Rad).

## 3 Results

### 3.1 PRMT5 inhibition induces functional ATM deficiency and rewires DDR signaling in PDAC cells

**FIGURE 1:**
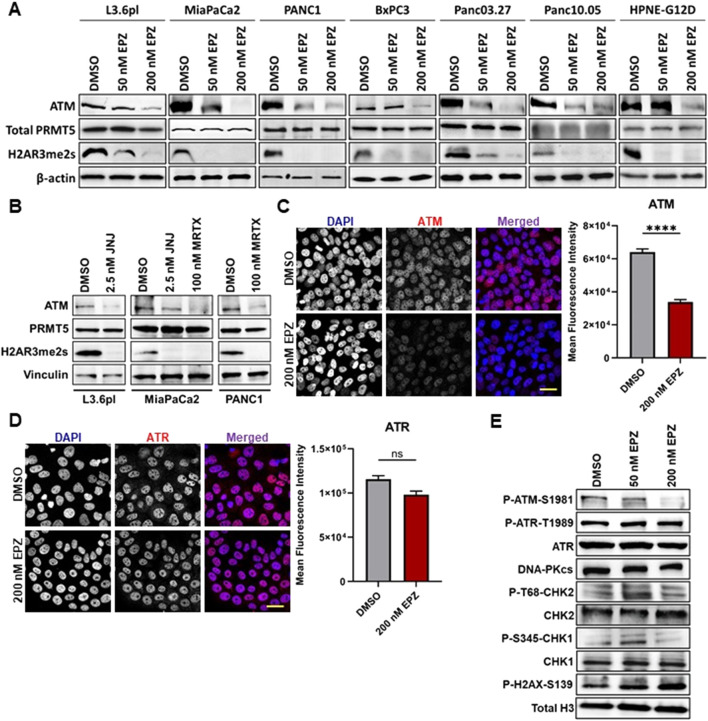
PRMT5 inhibition produces a universal ATM reduction across PDAC models, establishing a functional ATM-deficient phenotype. **(A)** Western blot analysis of ATM in various PDAC cell lines treated with vehicle (DMSO) or 50–200 nM EPZ015938 (EPZ) for 5 days. Total PRMT5 levels remain unchanged during treatment. The PRMT5-dependent mark H2AR3me2s confirms on-target PRMT5 inhibition. β-actin was used as a loading control. Representative blots from 3 biological replicates are shown. **(B)** Protein expression of ATM for the vehicle (DMSO) control, 2.5 nM JNJ64619178, or 100 nM MRTX9768 was quantified via Western blot analysis. Representative blots are shown. **(C,D)** Immunofluorescence of **(C)** ATM and **(D)** ATR in L3.6 PL cells after 96 h of treatment with vehicle (DMSO) or 200 nM EPZ. Representative images are shown (×20 magnification; scale bar = 40 µm). Quantification of mean fluorescence intensity in positive nuclei is plotted as Mean ± SEM. (n = 3, ≥50 cells per replicate; ****p ≤ 0.0001; Kolmogorov-Smirnov test). **(E)** Western blot analysis of related DDR proteins in L3.6 PL cells treated with vehicle or 50–200 nM EPZ. Total H3 is loading control. Representative blots from 3 biological replicates are shown.

We first examined whether PRMT5 inhibition alters the DNA damage response in PDAC cells by assessing ATM abundance. Across all PDAC lines tested, treatment with the PRMT5 inhibitor EPZ015938 (EPZ) consistently reduced total ATM protein levels (Figure 1A). PRMT5 levels remained stable, and the PRMT5-dependent histone mark H2AR3me2s decreased, confirming on-target enzymatic inhibition. This reduction in ATM levels is consistent with prior observations in other tumor types (Vlummens et al., 2022; Carter et al., 2023), suggesting a conserved functional interaction between PRMT5 and ATM. We refer to this drug-induced state as PRMT5-dependent functional ATM insufficiency. This finding is translationally relevant, as genomic analyses from our laboratory and others have shown that ATM haploinsufficiency due to mutation or copy-number loss occurs in a substantial subset of patients with PDAC (Roberts et al., 2015; Makohon-Moore and Iacobuzio-Donahue, 2016; Petersen, 2016; Chaffee et al., 2017; Raphael et al., 2017; Shindo et al., 2017; Zimmermann et al., 2021). Thus, pharmacologic PRMT5 inhibition induces a functional state that mirrors a clinically relevant genetic vulnerability. To assess the generality of this response, we evaluated two additional, structurally distinct PRMT5 inhibitors, JNJ64619178 and MRTX9768 (Figure 1B). Both compounds significantly reduced ATM protein levels compared to vehicle controls. Notably, MRTX9768 is an MTA-cooperative inhibitor active in MTAP-deleted contexts, further supporting that ATM reduction is a shared response to PRMT5 inhibition across inhibitor classes and genetic backgrounds.

Having established that ATM reduction is a consistent consequence of PRMT5 inhibition across PDAC models, we next examined this effect in greater detail in the L3.6 PL cell line. EPZ treatment markedly decreased nuclear ATM staining, with a 50% reduction in mean fluorescence intensity by immunofluorescence analysis (Figure 1C). In contrast, ATR signal intensity remained relatively unchanged (Figure 1D), indicating that PRMT5 inhibition in PDAC cells selectively diminishes ATM levels without affecting ATR. We next investigated canonical downstream ATM signaling and compensatory DDR pathways involving ATR and DNA-PK. As expected, phosphorylated ATM (S1981) decreased following EPZ treatment, consistent with reduced ATM pathway activation (Figure 1E). Total ATR and DNA-PK protein levels remained unchanged, indicating that PRMT5 inhibition does not broadly alter core DDR kinases at these doses. At the checkpoint effector level, 50 nM EPZ treatment led to increased phosphorylation of CHK1 (P-S345) and CHK2 (T68) despite stable total CHK1 and CHK2 abundance, consistent with reports that ATR can signal to CHK2 when ATM activity is diminished (Joe et al., 2006; Pabla et al., 2008). At the 200 nM EPZ dose, the phospho-CHK1 and phospho-CHK2 responses were less pronounced, coinciding with greater ATM reduction and increased DNA damage at this higher concentration of PRMT5 inhibition. In parallel, phosphorylation of H2A.X at S139 increased across doses, reflecting the accumulation of DNA damage. Together, these findings indicate that PRMT5 inhibition reduces ATM protein levels without affecting other DDR signaling, suggesting a functional shift toward reliance on ATR-dependent DDR regulation in PDAC cells.

### 3.2 Combined PRMT5 and CHK1 inhibition synergistically suppresses PDAC cell growth and survival

**FIGURE 2:**
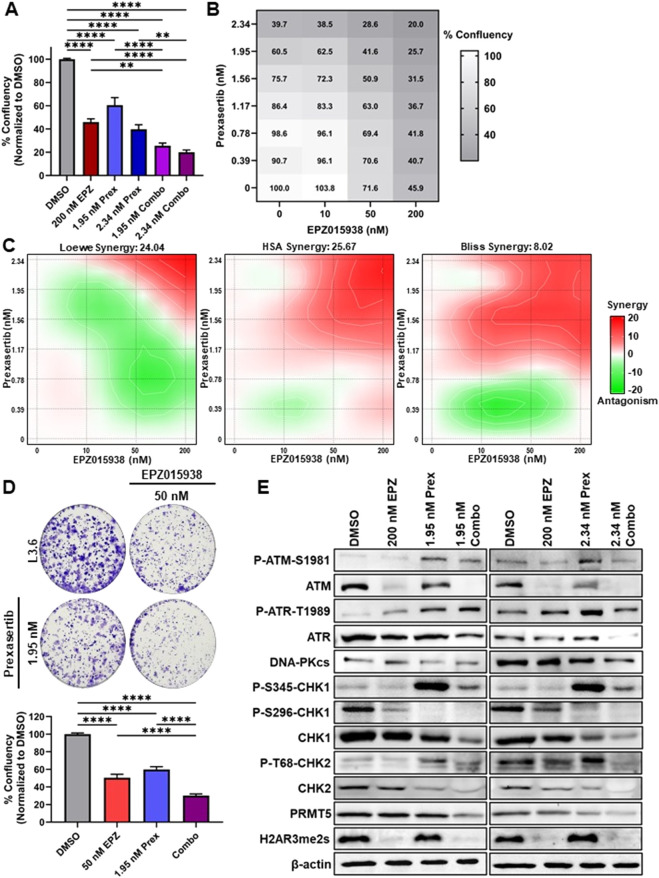
Dual PRMT5 and CHK1 inhibition synergistically impairs PDAC cell growth. L3.6 PL cells were treated with increasing concentrations of the PRMT5 inhibitor EPZ015938 (EPZ; 10–200 nM) and the CHK1 inhibitor prexasertib (Prex; 0.39–2.34 nM). **(A)** Cell viability was measured after 120 h using the IncuCyte SX5 Live Cell Analysis system. Data represent mean ± SEM. (n = 3; **p ≤ 0.01, ***p ≤ 0.005, and ****p ≤ 0.001; one-way ANOVA with Tukey’s *post hoc*). **(B)** Summary of the confluency values at 120 h used for synergy modeling. **(C)** Pharmacological interaction maps generated with SynergyFinder3.0 showing the combined effects of EPZ015938 and prexasertib at 120 h. Regions of synergism are shown in red. **(D)** Clonogenic survival assays of L3.6 PL cells treated with 50 nM EPZ015938 and 2.34 nM prexasertib, alone or in combination, for 11 days. Representative images and quantified colony area are shown. Data represent mean ± SD. (n = 10; ****p ≤ 0.001; one-way ANOVA with Tukey’s *post hoc*). **(E)** Western blot analysis of ATM and associated DNA damage response proteins following treatment with vehicle (DMSO), 200 nM EPZ, 2.34 nM Prex, or their combination. Total PRMT5 levels remain unchanged during treatment. The PRMT5-dependent mark H2AR3me2s confirms PRMT5 inhibition, and the auto-phosphorylation mark of CHK1 at S296 confirms on-target CHK1 inhibition. Total H3 and β-actin were used as loading controls. Representative blots from 3 replicates are shown.

Given these signaling changes, we next tested whether concurrent CHK1 targeting would potentiate the therapeutic impact of PRMT5 inhibition in PDAC cells. For this purpose, we treated L3.6 PL cells with EPZ and the CHK1 inhibitor prexasertib, either alone or in combination, and monitored cell growth over 5 days using live-cell imaging. Concentrations of EPZ and prexasertib selected for combination treatment were optimized from initial dose curves (Supplementary Figure S1). EPZ alone reduced confluency by 54.08% at 200 nM (p ≤ 0.0001), while prexasertib alone reduced confluency by 39.48% and 60.3% at 1.95 nM and 2.34 nM, respectively (p ≤ 0.0001; Figure 2A). Notably, the combination of 200 nM EPZ with 1.95 nM or 2.34 nM prexasertib further decreased cell growth by 74.34% and 79.95% (p ≤ 0.0001; Figure 2A). To characterize the interaction between PRMT5 and CHK1 inhibition, we modeled cell growth responses across a concentration matrix (Figure 2B). In quantitative synergy modeling, Loewe and HSA scores above +10 are typically considered indicative of strong synergy, while Bliss scores above +5 reflect meaningful positive deviation from independence. The analysis revealed a synergistic interaction between EPZ and prexasertib at concentrations ≥50 nM and ≥1.17 nM, respectively, with a peak Loewe model synergy score of 24.04, HSA score of 25.67, and Bliss score of 8.02 (Figure 2C). These values, derived from orthogonal reference models, indicate model-consistent, strong synergy between PRMT5 and CHK1 inhibition. To determine the impact of long-term treatment, we next performed colony formation assays, adjusting EPZ to 50 nM and maintaining prexasertib at 1.95 nM to account for the extended duration. Combination treatment led to an 86.1% reduction in colony formation (p ≤ 0.001; Figure 2D), confirming a durable, synergistic decrease in clonogenic survival.

**FIGURE 3:**
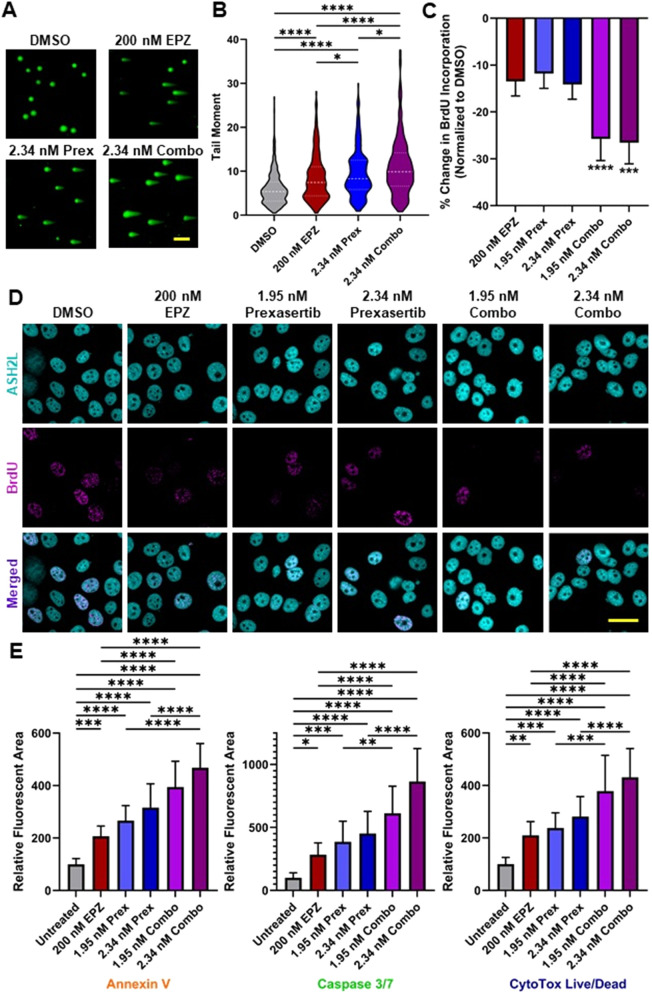
Co-inhibition of PRMT5 and CHK1 increases DSB accumulation and induces apoptotic cell death in PDAC cells. L3.6 PL cells were treated with 200 nM EPZ015938 and 1.95–2.34 nM prexasertib, alone or in combination. **(A,B)** Neutral comet assay detecting DSBs in L3.6 PL cells after 48-h treatment. Representative fields are shown (×4 magnification; scale bar = 100 µm). Violin plots depict the distribution of tail moments across treatments. (n = 4; ***p ≤ 0.005, and ****p ≤ 0.001; log-transformed one-way ANOVA with Tukey’s *post hoc*). **(C,D)** BrdU proliferation measuring DNA synthesis following 72-h drug exposure and 1-h BrdU pulse. ASH2L was used as a nuclear marker to detect the total number of cells. Representative images (×60 magnification; scale bar = 40 µm) and quantification of change in BrdU-positive nuclei are shown as mean ± SEM. (n = 4; ***p ≤ 0.005 and ****p ≤ 0.0001; Kruskal–Wallis H-test with Dunn’s *post hoc*). **(E)** Live-cell cell death assays using IncuCyte SX5 imaging. Cells were treated for 96 h in the presence of Caspase 3/7 green, Annexin V red, and CytoTox NIR dyes, and fluorescence was quantified over time. Caspase 3/7, Annexin V, and total cell death levels after 96 h are shown as mean ± SEM. (n = 3; *p ≤ 0.05, **p ≤ 0.01, ***p ≤ 0.005, and ****p ≤ 0.001; one-way ANOVA with Tukey’s *post hoc*).

Subsequently, we examined potential mechanisms underlying this synergistic effect. Western blot analysis revealed increased P-S345-CHK1 with prexasertib alone and in combination, consistent with checkpoint activation, while P-S296-CHK1 decreased under both conditions, confirming on-target inhibition (Figure 2E). CHK2 phosphorylation at T68 and total CHK2 protein levels were reduced with prexasertib alone and in combination, suggesting suppression of CHK2 signaling. PRMT5 protein levels remained unchanged, whereas its methylation mark H2AR3me2s was diminished in EPZ-treated and combination samples, confirming on-target PRMT5 inhibition. Thus, dual PRMT5-CHK1 inhibition disrupts checkpoint signaling at multiple nodes, attenuating ATM-CHK2 signaling and impairing CHK1 activity while leaving upstream ATR activation intact, consistent with a breakdown of coordinated DDR control. We next assessed whether co-inhibition increased DNA damage accumulation. Because the neutral comet assay specifically detects double-strand DNA breaks (DSBs), we used this approach to quantify cumulative damage across treatment conditions. In vehicle-treated L3.6 PL cells, the geometric mean tail moment was 4.994, while treatment with EPZ alone increased the tail moment to 7.031 (p ≤ 0.0001) (Figures 3A,B; Supplementary Figure S2), indicating accumulation of DSBs. Prexasertib alone had a geometric mean tail moment of 7.955 (p ≤ 0.0001), and the combination treatment amplified the effect, with a geometric mean tail moment reaching 9.114 (p ≤ 0.0001). Together, these findings demonstrate that concurrent PRMT5 and CHK1 inhibition leads to greater accumulation of DSBs than either agent alone, consistent with the changes in DDR signaling detected by Western blot. To determine whether this accumulation of DNA damage was accompanied by altered cell-cycle progression, we next examined DNA synthesis by BrdU incorporation. Relative to vehicle-treated L3.6 PL cells, the proportion of BrdU-positive nuclei decreased modestly but not significantly following EPZ treatment (−13.48%) or prexasertib alone at 1.95 nM (−11.81%) and 2.34 nM (−14.14%). The reduction was significantly greater in the combination groups, down 25.75% for 200 nM EPZ +1.95 nM prexasertib (p ≤ 0.0001) and 26.57% for 200 nM EPZ +2.34 nM prexasertib (p ≤ 0.005). These results indicate that dual PRMT5-CHK1 inhibition impairs S-phase progression more effectively than either agent alone. Lastly, we evaluated whether this proliferative arrest was accompanied by apoptosis in L3.6 PL cells. Using live-cell imaging with multiplex fluorescence markers for Annexin V (early apoptosis), Caspase 3/7 (late apoptosis), and total cell death, we found that cell death increased 2.10-fold (p ≤ 0.01 vs. DMSO) when treated with 200 nM EPZ, 2.38-fold (p ≤ 0.005 vs. DMSO) when treated with 1.95 nM prexasertib, and 2.81-fold (p ≤ 0.0001 vs. DMSO) when treated with 2.34 nM prexasertib (Figure 3E). Comparatively, when co-treated with 200 nM EPZ and 1.95 nM prexasertib, cell death increased 3.79-fold (p ≤ 0.0001 vs. DMSO), and 4.31-fold (p ≤ 0.0001 vs. DMSO) when co-treated with 200 nM EPZ and 2.34 nM prexasertib (Figure 3E; Supplementary Figure S2). Both Annexin V and Caspase 3/7 signaling were significantly elevated across treatment conditions, with maximal responses in combination-treated cells, consistent with induction of apoptosis. With 200 nM EPZ, Annexin V increased 2.07-fold (p ≤ 0.005) and Caspase 3/7 increased 2.84-fold (p ≤ 0.05). For single-agent prexasertib, the 1.95 nM dose increased the Annexin V 2.66-fold (p ≤ 0.0001) and Caspase 3/7 3.86-fold (p ≤ 0.005), whereas 2.34 nM prexasertib increased Annexin V 3.16-fold (p ≤ 0.0001) and Caspase 3/7 4.52-fold (p ≤ 0.0001). Notably, combination treatment further amplified these effects, with 200 nM EPZ plus 1.95 nM prexasertib increasing Annexin V 3.95-fold and Caspase 3/7 6.13-fold, and 200 nM EPZ plus 2.34 nM prexasertib increasing Annexin V 4.68-fold and Caspase 3/7 8.65-fold (all p ≤ 0.0001) (Figure 3E; Supplementary Figure S2). Combined, these results demonstrate that combined inhibition of PRMT5 and CHK1 not only intensifies DNA damage accumulation but also drives apoptotic cell death, consistent with the synergistic reduction in cell growth and colony formation.

### 3.3 Dual targeting of PRMT5 and CHK1 alters gene expression networks governing DNA repair, cell-cycle progression, and cell death in PDAC cells

**FIGURE 4:**
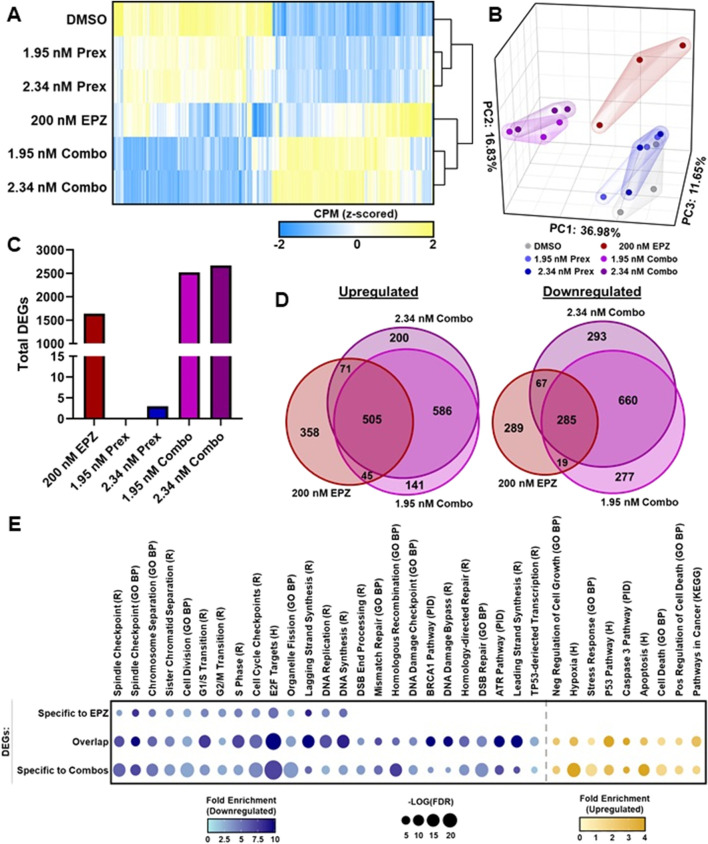
RNA-seq reveals that combined PRMT5 and CHK1 inhibition reshapes the transcriptional landscape to promote DNA damage, cell cycle arrest, and apoptosis. L3.6 PL cells were treated with 200 nM EPZ015938 and 1.95–2.34 nM prexasertib, alone or in combination, for 5 days prior to RNA extraction and sequencing. **(A)** Heatmap showing Z-scored counts per million for averaged replicate DEGs (n = 3/condition; FC ≥±2; FDR<0.05). **(B)** 3D PCA plot representing the variance among treatment groups. **(C)** Total number of DEGs per treatment group (FC ≥ ±2; FDR <0.05). **(D)** Euler plots displaying overlap of upregulated and downregulated DEGs between EPZ and combination (1.95 nM and 2.34 nM prexasertib) treatments. **(E)** Functional enrichment analysis of DEGs categorized as unique to EPZ, unique to combination treatments, or shared between both. Significantly enriched pathways were identified using KEGG (KEGG), Gene Ontology- Biological Processes (GO BP), Hallmark (H), Protein Interactome (PID), and Reactome (R) databases (q < 0.05).

Given the *in vitro* efficacy of combined PRMT5 and CHK1 inhibition and the known epigenetic functions of PRMT5, we next examined the transcriptional consequences of dual inhibition in L3.6 PL cells. Cells were treated with 200 nM EPZ015938 and 1.95–2.34 nM prexasertib, alone or in combination, for 5 days prior to RNA sequencing. Across all treatment groups, 3797 genes were identified as differentially expressed (DEGs; fold change≥±2, FDR<0.05) relative to vehicle control (Figure 4A). Heatmap visualization of DEGs revealed distinct gene clustering among treatment groups, which was corroborated by principal component analysis (PCA). PCA showed that prexasertib-treated samples clustered closely with vehicle controls, suggesting minimal transcriptional perturbation with prexasertib alone (Figure 4B). In contrast, EPZ-treated and combination-treated samples clustered together, indicating shared and potentially cooperative effects. Notably, a summary of total DEGs confirmed that prexasertib alone produced few transcriptional changes, with >99% of DEGs arising from the EPZ or combination treatments (Figure 4C; Supplementary Figure S3). Comparative analysis identified 979 genes upregulated by EPZ alone, whereas the combination treatment induced an additional 927 genes that were upregulated exclusively in co-treated samples (Figure 4D). Similarly, EPZ alone downregulated 660 genes, and the combination downregulated an additional 1230 genes (Figure 4D). The higher level of transcriptional changes with the combination, along with the minimal effect of prexasertib alone, suggests that PRMT5 inhibition establishes a permissive epigenetic landscape, making gene networks more responsive to CHK1 pathway disruption. For functional interpretation, we performed pathway enrichment analysis using the Molecular Signatures Database (MSigDB) and visualized results as bubble plots depicting pathway significance and normalized enrichment scores (Figure 4E). Genes were categorized as unique to EPZ, unique to the combination treatment, or shared between both. PRMT5 inhibition alone markedly reshaped the transcriptome, whereas co-inhibition with CHK1 enhanced these effects and activated distinct, additional transcriptional programs. As shown in Figure 4D, combination-treated cells exhibited a greater number of both upregulated and downregulated genes compared with EPZ alone. Correspondingly, pathway enrichment analysis (Figure 4E; Supplementary Table S2) revealed amplification of shared pathways and involvement of novel ones, with combination treatment consistently affecting additional genes within overlapping networks. Enriched pathways reflected the biological phenotypes observed in earlier assays, including downregulation of cell-cycle progression and DNA replication/repair genes and upregulation of cell death-associated processes (Figure 4E; Supplementary Table S2). To validate these findings, we performed an RT-qPCR array targeting DNA damage signaling pathway genes, which corroborated the RNA-seq patterns (Supplementary Figure S4; Supplementary Tables S3, S4). Collectively, these molecular signatures demonstrate that while PRMT5 inhibition alone suppresses proliferative and repair-associated transcriptional programs, combined PRMT5 and CHK1 inhibition drives a broader and more pronounced transcriptional reprogramming that enhances DNA damage accumulation, enforces cell-cycle arrest, and promotes cell death.

### 3.4 Combined PRMT5 and CHK1 inhibition enhances anti-tumor efficacy and extends survival in PDAC xenograft models

**FIGURE 5:**
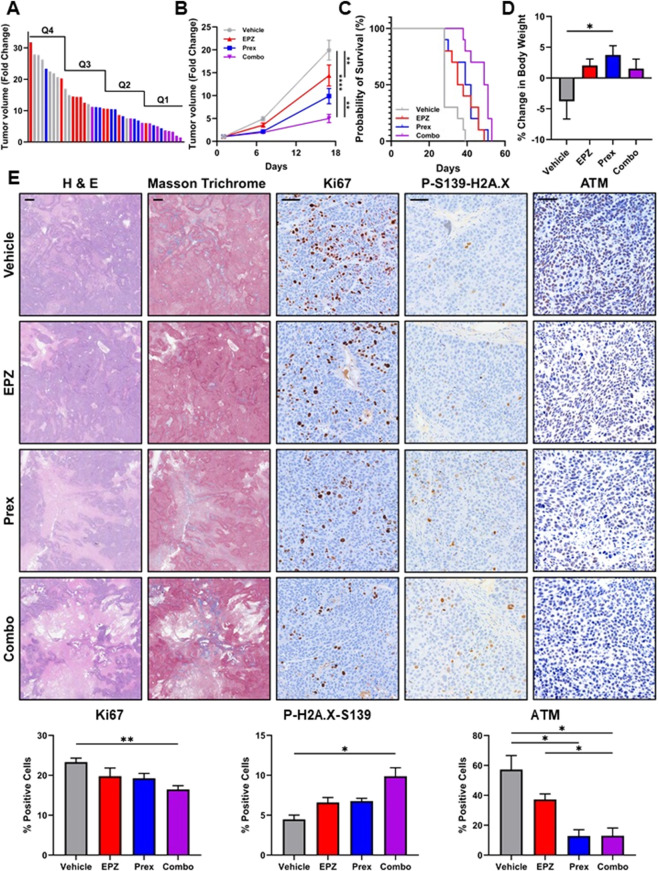
PRMT5 and CHK1 co-inhibition significantly reduces tumor growth and prolongs survival *in vivo*. L3.6 PL cells were injected subcutaneously into female athymic nude NU/J mice aged 6–8 weeks. Once tumor volumes reached ∼100 mm^3^, animals were randomized into four treatment groups (vehicle control, 50 mg/kg EPZ015938, 2 mg/kg prexasertib, or their combination). The experiment was performed twice independently with n = 5 mice per group per replicate (total n = 10 per group). **(A,B)** Tumor growth kinetics and endpoint tumor volumes relative to initial size over 17 days of treatment. (n = 10 per group; *p ≤ 0.05, **p ≤ 0.01, ***p ≤ 0.001; one-way ANOVA with Tukey’s *post hoc*). **(C)** Kaplan-Meier survival analysis showing prolonged survival in combination-treated mice compared with controls and single-agent treatments (n = 10 per group; log-rank test; p ≤ 0.001). **(D)** Mean change in body weight over the duration of treatment for each group. Data are shown as mean ± SEM. (*p ≤ 0.05; one-way ANOVA with Tukey’s *post hoc* test). **(E)** Representative histological and immunohistochemical images of tumors stained with hematoxylin and eosin (H,E), Masson trichrome (×5 magnification; scale bar = 100 µm), or Hematoxylin-DAB IHC for Ki67, γH2A.X, and ATM (×20 magnification; scale bar = 100 µm). The percentage of tumor cells positive for Ki67, γH2A.X, and ATM is shown. (mean ± SEM; n = 5; *p ≤ 0.05, **p ≤ 0.01; Brown-Forsythe and Welch ANOVA with Dunnett’s *post hoc*).

Subsequently, we evaluated the therapeutic efficacy of combined PRMT5 and CHK1 inhibition *in vivo* using subcutaneous L3.6 PL xenografts. Once tumors reached 100mm^3^, mice were randomized into four groups to receive vehicle, EPZ015938, prexasertib, or the combination. Analysis of tumor volume FC distribution showed that most combination-treated tumors (90%) fell within the two lowest quartiles, with 70% residing in the lowest quartile, corresponding to the smallest tumor volumes (Figure 5A). A one-way ANOVA showed a significant overall effect of treatment on tumor growth at day 17 (p < 0.0001). Post-hoc comparisons revealed that EPZ015938 significantly reduced tumor growth compared with the control group (p ≤ 0.01; 9.8 ± 1.6 FC vs. 19.9 ± 2.1 FC), whereas prexasertib alone did not (p > 0.05; 14.4 ± 2.2 FC vs. 19.9 ± 2.1 FC). The combination treatment produced the strongest effect, reducing tumor growth to 5.0 ± 0.9 FC (p ≤ 0.0001 vs. control) and showing greater inhibition than either individual treatment (p ≤ 0.05 vs. prexasertib; p ≤ 0.01 vs. EPZ015938; Figure 5B). Furthermore, combination-treated animals exhibited significantly prolonged survival, with a median survival of 50 days compared to 28 days for controls, 36.5 days for EPZ015938 alone, and 40.5 days for prexasertib alone (p ≤ 0.001; Figure 5C). All treatment groups maintained body weight within 5% of baseline throughout the study. Treated mice exhibited slight weight gains (1.5%–3.8%), whereas untreated controls lost an average of 3.8% (Figure 5D), indicating that the combination treatment did not adversely affect body weight. Histopathologic analysis of resected tumors revealed reduced overall tumor size and increased necrosis in combination-treated mice, along with dense peritumoral fibrosis evident by enhanced blue fiber staining on Masson’s trichrome sections. Immunohistochemical analysis showed a significant decrease in the proliferation marker Ki67 in combination-treated tumors (16.46% ± 0.96% vs. 23.32% ± 0.99% in controls, p ≤ 0.01). The DNA damage marker γH2A.X (P-S139-H2A.X) was elevated (9.88% ± 1.05% vs. 4.478% ± 0.54% for controls, p ≤ 0.05), while total ATM staining was substantially reduced (12.98% ± 5.14% vs. 57.26% ± 9.36% in controls, p ≤ 0.05; Figure 5E). These *in vivo* findings mirror our *in vitro* results, demonstrating that combined PRMT5 and CHK1 inhibition amplifies DNA damage, suppresses proliferation, and reduces ATM levels in PDAC cells. Together, these data establish that dual inhibition of PRMT5 and CHK1 represents a promising, well-tolerated therapeutic strategy that limits tumor growth and prolongs survival *in vivo*.

## 4 Discussion

In this study, we apply mechanistic insights into epigenomic and DNA repair pathway interactions to rationally design a new combination strategy for PDAC. Our approach identifies a therapeutic opportunity resulting from PRMT5 inhibition, which induces a state of functional ATM deficiency and increases reliance on ATR-CHK1 signaling. By simultaneously inhibiting CHK1, we find a synergistic effect with reduced cell growth, increased apoptosis, alterations in DNA repair and survival gene networks, and improved *in vivo* anti-tumor efficacy and animal survival. Importantly, these rationally designed combinations of epigenomic and checkpoint inhibitors not only reveal new vulnerabilities in PDAC but may also limit the overall toxicity concerns associated with the clinical use of epigenomic drugs as monotherapies. Following, we examine the mechanistic and translational implications of this combination strategy, discussing how PRMT5-dependent ATM modulation influences DDR regulation and guides therapeutic targeting in PDAC.

We report that inhibiting PRMT5 disrupts ATM-dependent signaling, creating a cellular environment where ATR-CHK1 activity becomes the primary mechanism for maintaining DNA integrity and cell survival. Across various PDAC models and using different PRMT5 inhibitors, we consistently detect reductions in both total and phospho-ATM. ATM is a crucial kinase in PDAC, as our laboratory and others have identified a high rate of ATM mutations in patients presenting to our clinics (Russell et al., 2015; Armstrong et al., 2019; Gower et al., 2021; Zimmermann et al., 2021). Furthermore, tumors with ATM loss exhibit distinct biological features and treatment responses (Armstrong et al., 2019; Gower et al., 2021; Hannan et al., 2021), underscoring the relevance of this pathway and its potential as a therapeutic target in PDAC. Notably, our RNA-seq and pathway-specific RT-qPCR analyses reveal no significant changes in total ATM mRNA levels following PRMT5 inhibition. This suggests that the observed decrease in ATM protein likely reflects post-transcriptional or post-translational regulation, potentially involving altered splicing, changes in protein stability, or enhanced degradation rather than transcriptional repression. Although prior studies have reported transcriptional regulation of *ATM* by PRMT5 in other contexts (Hamard et al., 2018; Vlummens et al., 2022; Carter et al., 2023; Gillespie et al., 2024), our results are consistent with recent genome-wide CRISPR-based studies in a different model showing that PRMT5 influences ATM protein abundance independent of transcript levels (Huang et al., 2023). Defining the precise mechanism by which PRMT5 inhibition reduces ATM protein levels in PDAC will be an important focus of future studies. At the molecular level, the reduced ATM signaling shifts the DNA damage response toward ATR-CHK1-mediated signaling, creating a compensatory yet targetable survival pathway. At the cellular level, this shift enhances sensitivity to CHK1 inhibition, as evidenced by synergistic suppression of PDAC cell growth, increased apoptosis, and greater DNA damage. Drug-dose modeling confirmed that this interaction is synergistic rather than additive within a specific concentration range. We also recognize that our conclusions are based on a defined panel of PDAC models that, while molecularly diverse, cannot fully capture the full heterogeneity of human PDAC, underscoring the need for validation in additional patient-derived organoids and *in vivo* models. Together, these findings outline a mechanistic cascade in which loss of PRMT5 activity destabilizes ATM and disrupts checkpoint balance, a change that is reflected in the transcriptional alterations accompanying dual pathway inhibition.

Previous studies have shown that PRMT5 activity affects the expression of several genes involved in DNA repair and replication through chromatin and splicing mechanisms across different cancer types (Owens et al., 2020; Carter et al., 2023; Gillespie et al., 2024). In PDAC, our RNA-seq data support these findings. They reveal that inhibiting PRMT5 alone alters the transcriptome, with coordinated downregulation of cell-cycle and DNA-repair networks and activation of apoptosis pathways. However, these transcriptional effects are even stronger when CHK1 is targeted concurrently. Cells treated with both inhibitors exhibit an expanded DEG signature compared with those solely treated with PRMT5 inhibition. Notably, targeting CHK1 alone produces only minor changes in gene expression. This suggests that inhibiting PRMT5 already establishes a chromatin landscape in which CHK1 signaling exerts greater regulatory influence on transcriptional programs. The resulting disruption of DNA-repair capacity and signaling impairs cell survival, thereby defining the mechanistic basis for the synergistic interaction between PRMT5 and CHK1 inhibition in PDAC. However, the increased CHK1 dependence following PRMT5 inhibition likely reflects not only reduced ATM function but also broader PRMT5-regulated processes, including effects on RNA splicing, chromatin regulation, and DNA repair gene networks. A more complete understanding of the relative contributions of these processes, as well as potential off-target and long-term effects of dual PRMT5/CHK1 inhibition, will require additional focused studies.

Importantly, the mechanisms identified in cellular studies were recapitulated *in vivo*. In PDAC xenografts, inhibiting both PRMT5 and CHK1 significantly suppressed tumor growth, induced greater DNA damage, reduced proliferative activity, and decreased ATM levels. Histological analyses confirmed higher phospho-H2A.X levels and lower Ki-67 staining, consistent with DNA damage accumulation and reduced cell proliferation. Together, these tumor responses support effective target engagement *in vivo* and are consistent with the molecular and cellular mechanisms observed *in vitro*. Given the dense stromal architecture of PDAC, microenvironmental factors may influence drug delivery and response to dual PRMT5/CHK1 inhibition, and additional studies in complementary models will help refine these results. Similar combination strategies have been identified in other cancer types, such as mantle cell lymphoma, where PRMT5 inhibition synergizes with ATR or CDK4 blockade, particularly in ATM- or TP53-mutant cases (Che et al., 2023). Our results extend this rationale to PDAC, demonstrating that PRMT5-mediated attenuation of ATM function provides an opportunity to enhance sensitivity to CHK1 targeting. This consistency across tumor types reinforces the concept that PRMT5-dependent alterations in DDR signaling constitute a common weakness amenable to combination strategies.

Several PRMT5 inhibitors are progressing in clinical trials, indicating rapid advancements toward more effective and better-tolerated therapeutic strategies. The inhibitors used in this study, EPZ015938, JNJ-64619178, and MRTX9768, represent different mechanistic classes that are similar to or earlier than current clinical candidates (Hu and Chen, 2024; Kaganovski et al., 2025). Consistent effects with three mechanistically distinct PRMT5 inhibitors in multiple PDAC models support the likelihood that ATM downregulation is a class effect of PRMT5 inhibition, although evaluation of additional clinical candidates will further refine the translational applicability of these findings. EPZ015938 is a research analog of the first-generation GSK3326595 (EPZ015666), while JNJ-64619178 (Onametostat) has advanced into phase I/II evaluation for adenoid cystic carcinoma and hematological malignancies (Vieito et al., 2023; Kaganovski et al., 2025). MRTX9768 is an MTA-cooperative inhibitor related to the clinical compound MRTX1719, which demonstrates strong synthetic lethality in MTAP-deleted tumors and has improved tolerability (Engstrom et al., 2023). Other SAM-competitive inhibitors, such as BMS-986504, AZD3470, and LNP7457, and MTA-cooperative agents, including TNG462 and TNG908, are being tested in MTAP-deficient solid tumors with a focus on biomarker-guided patient selection and minimizing hematologic toxicity (Hu and Chen, 2024; Kaganovski et al., 2025). Similarly, CHK1 inhibitors, particularly prexasertib, are currently in clinical trials for cancers with high replication stress, including platinum-resistant ovarian and triple-negative breast cancers, showing durable responses and manageable toxicity both as monotherapy and when combined with DNA-damaging agents (Giudice et al., 2024; Jiang et al., 2024). Thus, the expanding clinical evaluation of PRMT5 and CHK1 inhibitors across multiple tumor types highlights the broader promise of this combination strategy and its particular relevance for PDAC. At the same time, this momentum underscores the need for continued investigation into how PRMT5 regulates ATM protein levels and DDR signaling, as well as further translational work to optimize this therapeutic approach. In particular, future studies will be important for defining optimal dosing regimens, assessing interactions with standard-of-care therapies such as chemotherapy or immunotherapy, and evaluating potential resistance mechanisms that may emerge with prolonged dual inhibition. In parallel, building on our findings, additional work will be required to develop and validate predictive biomarkers to prospectively identify patients most likely to benefit from dual PRMT5 and CHK1 inhibition in clinical practice. Collectively, these efforts will be essential for eventually translating this mechanistic framework into effective clinical implementation.

In summary, our work defines functional ATM deficiency as a mechanistic consequence of PRMT5 inhibition and demonstrates that this altered DDR signaling state can be therapeutically exploited by co-inhibiting CHK1 to enhance anti-tumor activity in PDAC. By linking epigenomic regulation to DDR network adaptation, these findings advance our understanding of how chromatin-modifying enzymes interface with DNA repair pathways. More broadly, this study offers a conceptual framework for rationally pairing epigenomic and DDR inhibitors to dismantle compensatory repair dependencies and improve therapeutic responses in PDAC.

## Data Availability

The data presented in the study are deposited in the Gene Expression Omnibus repository, accession number GSE310927.
