# Supplementary material for: PRMT5 inhibition triggers functional ATM deficiency and sensitizes pancreatic cancer to CHK1 blockade
Source: Front Cell Dev Biol. 2026 Mar 11;14:1748541. doi: 10.3389/fcell.2026.1748541 (PMC13013501; doi:10.3389/fcell.2026.1748541)
Supplement: Supplementary file 1 [file DataSheet1.pdf]

Supplementary Material

for

PRMT5 Inhibition Triggers Functional ATM Deficiency and Sensitizes Pancreatic Cancer to CHK1 Blockade

Dzikowski M[1], Pollin G[2,3], Gunia V[2,3], Butler S[2], Enkhtuul B[1], Gavina Chavez J[2], Zimmermann MT[2,4], Mathison AJ[2,3], Urrutia R[2,3,5], and Lomberk G[1,2,3]

1 Supplementary Figures and Tables

Supplementary Table 1: List of antibodies used

Antibody Name	Manufacturer	Catalogue Number	Dilution
ATM	abcam	ab32420	1:100
ATM	Cell Signaling	2873	1:1000
P-ATM-S1981	Invitrogen	MA5-32751	1:1000
ATR	Cell Signaling	2790	1:1000
P-ATR-T1989	Cell Signaling	30632S	1:1000
DNA-PKcs	Cell Signaling	12311	1:1000
P-S139-H2A.X	abcam	ab26350	1:1000
CHK1	Cell Signaling	2360	1:1000
CHK2	Cell Signaling	3440	1:1000
P-S296-CHK1	Novus Biologicals	NBP2-67711	1:1000
P-S345-CHK1	Cell Signaling	2348	1:1000
P-T68-CHK2	Cell Signaling	2197	1:1000
Histone H3	abcam	ab1791	1:1000
H2ARme2s	abcam	ab22397	1:1000
PRMT5	abcam	ab109451	1:1000
PRMT5	Cell Signaling	79998	1:1000
Ki67	abcam	ab16667	1:200
β-actin	abcam	ab184576	1:1000
BrdU	Invitrogen	B35128	1:1000
Lamin B1	abcam	ab16048	1:1000
ASH2L	Cell Signaling	5019	1:1000
Goat-anti-Mouse-HRP	Millipore	401215	1:2000
Goat-anti-Rabbit-HRP	Millipore	401315	1:2000
Goat-anti-Mouse-AlexaFluor546	Invitrogen	A11003	1:500-1:1000
Goat-anti-Rabbit-AlexaFluor488	Invitrogen	A11008	1:500-1:1000

Supplementary Table 2: Normalized expression scores (NES) for RNA-seq gene set enrichment analysis using KEGG (KEGG), Gene Ontology- Biological Processes (GO BP), Hallmark (H), Protein Interactome (PID), WikiPathways (WP), and Reactome (R) databases

Database	Pathway Name	Sample	Normalized Enrichment Score (NES)	FDR	Direction
PID	ATR Pathway	Combo Unique	6.650	1.92E-03	Down
PID	ATR Pathway	DEGs in Common	14.825	1.44E-05	Down
PID	BRCA1 Pathway	Combo Unique	3.618	1.87E-01	Down
PID	BRCA1 Pathway	DEGs in Common	11.523	7.95E-04	Down
GO BP	Cell Cycle	Combo Unique	2.334	1.51E-19	Down
GO BP	Cell Cycle	DEGs in Common	2.751	9.03E-12	Down
GO BP	Cell Cycle	EPZ Unique	1.779	6.09E-02	Down
R	Cell Cycle Checkpoints	Combo Unique	4.113	6.52E-13	Down
R	Cell Cycle Checkpoints	DEGs in Common	5.716	9.71E-10	Down
R	Cell Cycle Checkpoints	EPZ Unique	3.783	8.21E-03	Down
GO BP	Cell Division	Combo Unique	2.901	5.67E-12	Down
GO BP	Cell Division	DEGs in Common	2.843	2.50E-04	Down
GO BP	Cell Division	EPZ Unique	2.779	2.97E-02	Down
GO BP	Chromosome Organization	Combo Unique	2.281	1.17E-10	Down
GO BP	Chromosome Organization	DEGs in Common	2.739	9.30E-07	Down
GO BP	Chromosome Organization	EPZ Unique	1.877	1.11E-01	Down
GO BP	Chromosome Separation	Combo Unique	4.975	6.51E-08	Down
GO BP	Chromosome Separation	DEGs in Common	5.762	5.92E-04	Down
GO BP	Chromosome Separation	EPZ Unique	4.928	6.09E-02	Down
R	DNA Damage Bypass	Combo Unique	3.204	1.99E-01	Down
R	DNA Damage Bypass	DEGs in Common	9.795	5.18E-04	Down
GO BP	DNA Damage Checkpoint	Combo Unique	3.259	1.64E-03	Down
GO BP	DNA Damage Checkpoint	DEGs in Common	4.152	2.12E-02	Down
WP	DNA Damage Response	Combo Unique	3.166	1.93E-01	Down
WP	DNA Damage Response	DEGs in Common	5.762	2.68E-02	Down
WP	DNA Repair Pathways	Combo Unique	5.169	3.31E-07	Down
WP	DNA Repair Pathways	DEGs in Common	8.560	4.30E-07	Down
WP	DNA Repair Pathways	EPZ Unique	4.023	1.26E-01	Down
R	DNA Replication	Combo Unique	2.455	1.17E-02	Down
R	DNA Replication	DEGs in Common	6.622	1.91E-08	Down
R	DNA Replication	EPZ Unique	4.045	2.30E-02	Down
R	DNA Synthesis	Combo Unique	3.226	1.46E-03	Down
R	DNA Synthesis	DEGs in Common	8.768	4.64E-09	Down
R	DNA Synthesis	EPZ Unique	5.356	1.58E-02	Down
R	DSB End Processing	Combo Unique	3.417	4.00E-03	Down

R	DSB End Processing	DEGs in Common	3.628	8.01E-02	Down
GO BP	DSB Repair	Combo Unique	3.844	1.39E-10	Down
GO BP	DSB Repair	DEGs in Common	4.270	6.70E-05	Down
H	E2F Targets	Combo Unique	7.227	1.90E-25	Down
H	E2F Targets	DEGs in Common	10.187	3.27E-17	Down
H	E2F Targets	EPZ Unique	5.266	1.17E-04	Down
R	G1/S Transition	Combo Unique	3.618	8.75E-05	Down
R	G1/S Transition	DEGs in Common	7.682	6.54E-08	Down
R	G1/S Transition	EPZ Unique	4.380	2.94E-02	Down
R	G2/M Checkpoint	Combo Unique	3.245	8.06E-05	Down
H	G2/M Checkpoint	Combo Unique	4.152	9.60E-09	Down
R	G2/M Checkpoint	DEGs in Common	5.119	4.35E-05	Down
H	G2/M Checkpoint	DEGs in Common	8.620	2.99E-13	Down
R	G2/M Checkpoint	EPZ Unique	4.330	2.02E-02	Down
H	G2/M Checkpoint	EPZ Unique	5.266	1.17E-04	Down
R	G2/M Transition	Combo Unique	2.714	8.56E-04	Down
R	G2/M Transition	DEGs in Common	2.634	7.59E-02	Down
R	G2/M Transition	EPZ Unique	3.620	2.94E-02	Down
GO BP	Homologous Recombination	Combo Unique	7.469	9.62E-09	Down
GO BP	Homologous Recombination	DEGs in Common	5.597	4.30E-02	Down
R	Homology-directed Repair	Combo Unique	3.715	6.53E-05	Down
R	Homology-directed Repair	DEGs in Common	5.785	7.73E-05	Down
R	Lagging Strand Synthesis	Combo Unique	5.592	7.12E-02	Down
R	Lagging Strand Synthesis	DEGs in Common	28.494	1.26E-08	Down
R	Lagging Strand Synthesis	EPZ Unique	8.703	1.38E-01	Down
R	Leading Strand Synthesis	Combo Unique	6.590	1.19E-01	Down
R	Leading Strand Synthesis	DEGs in Common	33.583	4.71E-07	Down
GO BP	Mismatch Repair	Combo Unique	5.349	4.52E-03	Down
GO BP	Mismatch Repair	DEGs in Common	6.814	5.50E-02	Down
R	Mitosis	Combo Unique	3.089	1.35E-09	Down
R	Mitosis	DEGs in Common	2.846	1.54E-03	Down
R	Mitosis	EPZ Unique	2.455	5.30E-02	Down
GO BP	Negative Regulation of Cell Death	Combo Unique	3.742	1.03E-10	Down
GO BP	Negative Regulation of Cell Death	DEGs in Common	5.348	2.48E-08	Down
GO BP	Organelle Fission	Combo Unique	3.435	5.91E-15	Down
GO BP	Organelle Fission	DEGs in Common	3.196	1.04E-04	Down
GO BP	Organelle Fission	EPZ Unique	2.376	1.03E-01	Down
GO CC	Replisome	Combo Unique	5.126	9.78E-02	Down
GO CC	Replisome	DEGs in Common	16.325	3.45E-04	Down
R	S Phase	Combo Unique	3.125	3.28E-04	Down

R	S Phase	DEGs in Common	7.124	1.91E-08	Down
R	S Phase	EPZ Unique	4.608	1.63E-02	Down
R	Sister Chromatid Separation	Combo Unique	3.401	7.41E-06	Down
R	Sister Chromatid Separation	DEGs in Common	4.333	4.02E-04	Down
R	Sister Chromatid Separation	EPZ Unique	3.529	4.10E-02	Down
GO BP	Spindle Assembly Checkpoint Signaling	Combo Unique	7.048	2.09E-05	Down
GO BP	Spindle Assembly Checkpoint Signaling	DEGs in Common	9.795	1.55E-03	Down
GO BP	Spindle Assembly Checkpoint Signaling	EPZ Unique	7.978	8.46E-02	Down
R	Spindle Checkpoint	Combo Unique	5.783	3.64E-09	Down
R	Spindle Checkpoint	DEGs in Common	6.697	6.13E-05	Down
R	Spindle Checkpoint	EPZ Unique	3.273	1.92E-01	Down
GO CC	Telomeric Regions	Combo Unique	3.289	1.44E-04	Down
GO CC	Telomeric Regions	DEGs in Common	3.771	1.52E-02	Down
GO CC	Telomeric Regions	EPZ Unique	3.072	1.24E-01	Down
R	TP53-directed Transcriptional Regulation	Combo Unique	1.668	1.44E-01	Down
R	TP53-directed Transcriptional Regulation	DEGs in Common	2.772	4.14E-03	Down
H	Apoptosis	Combo Unique	4.055	1.34E-06	Up
H	Apoptosis	DEGs in Common	2.733	4.15E-02	Up
PID	Caspase 3 Pathway	Combo Unique	3.766	1.31E-01	Up
PID	Caspase 3 Pathway	DEGs in Common	6.346	8.38E-02	Up
GO BP	Cell Death	Combo Unique	1.618	7.62E-06	Up
GO BP	Cell Death	DEGs in Common	1.446	3.00E-02	Up
H	Hypoxia	Combo Unique	4.733	5.67E-11	Up
H	Hypoxia	DEGs in Common	3.025	5.56E-03	Up
GO BP	Negative Regulation of Cell Growth	Combo Unique	2.296	3.08E-02	Up
GO BP	Negative Regulation of Cell Growth	DEGs in Common	2.488	8.59E-02	Up
H	P53 Pathway	Combo Unique	2.938	2.40E-04	Up
H	P53 Pathway	DEGs in Common	4.125	2.64E-05	Up
KEGG	Pathways in Cancer	Combo Unique	1.786	2.23E-02	Up
KEGG	Pathways in Cancer	DEGs in Common	2.802	1.31E-04	Up
GO BP	Positive Regulation of Cell Death	Combo Unique	1.758	1.22E-02	Up
GO BP	Positive Regulation of Cell Death	DEGs in Common	1.777	7.50E-02	Up
GO BP	Stress Response	Combo Unique	1.468	2.87E-07	Up
GO BP	Stress Response	DEGs in Common	1.243	8.13E-02	Up

Supplementary Table 3: Fold Change and FDR values for key RNA-seq genes

	200 nM EPZ		1.95 nM Prex		2.34 nM Prex		1.95 nM Combo		2.34 nM Combo	
**Gene**	**Log2FC**	**FDR**	**Log2FC**	**FDR**	**Log2FC**	**FDR**	**Log2FC**	**FDR**	**Log2FC**	**FDR**
ABL1	-0.005	9.92E-01	0.194	9.87E-01	0.153	9.30E-01	0.642	5.94E-02	0.710	3.50E-02
APEX1	-0.507	1.28E-01	-0.247	9.85E-01	-0.365	7.46E-01	0.070	8.45E-01	-0.040	9.15E-01
ATM	1.242	2.59E-01	0.572	9.87E-01	0.707	8.79E-01	-0.904	4.21E-01	-0.879	4.39E-01
ATR	0.210	8.59E-01	0.316	9.93E-01	0.387	9.29E-01	-1.174	2.08E-01	-1.067	2.55E-01
ATRIP	-0.626	3.12E-02	-0.086	9.93E-01	-0.158	9.07E-01	-0.177	5.36E-01	-0.233	4.09E-01
ATRX	1.008	4.38E-01	0.263	9.93E-01	0.604	9.14E-01	-1.087	3.91E-01	-0.740	5.72E-01
BARD1	-0.860	1.71E-01	0.082	9.94E-01	0.056	9.85E-01	-1.640	7.36E-03	-1.564	9.07E-03
BAX	-0.393	3.10E-01	-0.207	9.87E-01	-0.210	9.03E-01	0.223	5.37E-01	0.107	7.89E-01
BBC3	-0.316	7.62E-01	0.086	9.95E-01	0.222	9.55E-01	0.529	5.19E-01	0.563	4.94E-01
BLM	-0.383	6.57E-01	-0.222	9.93E-01	-0.118	9.72E-01	-1.704	2.28E-02	-1.909	1.14E-02
BRCA1	-0.496	5.22E-01	0.016	9.99E-01	0.059	9.86E-01	-2.062	4.48E-03	-1.993	5.10E-03
BRIP1	-0.248	8.12E-01	0.125	9.94E-01	0.264	9.45E-01	-1.941	2.40E-02	-1.780	3.45E-02
CDC25A	-1.065	2.59E-04	-0.014	9.96E-01	-0.172	8.77E-01	-0.867	9.42E-04	-0.916	4.98E-04
CDC25C	-1.413	3.43E-04	-0.437	9.48E-01	-0.596	5.34E-01	-1.353	2.42E-04	-1.434	1.17E-04
CDK7	0.056	8.67E-01	0.056	9.93E-01	0.110	9.28E-01	0.106	7.02E-01	0.079	7.86E-01
CDKN1A	1.354	2.16E-02	0.953	8.52E-01	0.989	5.45E-01	2.642	2.57E-05	2.633	2.12E-05
CHEK1	-0.356	3.57E-01	-0.105	9.93E-01	-0.077	9.65E-01	-0.933	8.47E-03	-1.064	2.99E-03
CHEK2	-0.984	3.90E-03	-0.495	9.07E-01	-0.620	4.65E-01	-1.044	1.48E-03	-1.221	2.94E-04
CIB1	-0.492	3.50E-01	-0.107	9.93E-01	-0.207	9.28E-01	0.532	2.39E-01	0.435	3.50E-01
CRY1	-0.054	8.66E-01	0.180	9.87E-01	0.192	8.49E-01	-0.022	9.40E-01	0.120	6.43E-01
CSNK2A2	-0.138	6.33E-01	0.120	9.87E-01	0.030	9.80E-01	0.302	1.97E-01	0.399	8.07E-02
DDB1	-0.142	7.09E-01	0.064	9.93E-01	-0.089	9.54E-01	0.640	2.85E-02	0.664	2.20E-02
DDB2	-0.511	2.57E-01	-0.200	9.87E-01	-0.346	8.43E-01	-0.168	7.11E-01	-0.173	7.06E-01
DDIT3	-0.226	7.97E-01	0.620	9.69E-01	0.875	6.95E-01	0.720	2.68E-01	0.757	2.44E-01
ERCC1	-0.296	6.14E-01	0.123	9.93E-01	0.079	9.74E-01	0.798	8.46E-02	0.731	1.14E-01
ERCC2	-0.897	3.52E-02	-0.131	9.93E-01	-0.195	9.16E-01	-0.176	6.85E-01	-0.249	5.56E-01
EXO1	-1.098	3.22E-03	-0.009	9.99E-01	0.020	9.91E-01	-1.348	2.82E-04	-1.447	1.20E-04
FANCA	-1.263	4.39E-05	0.057	9.93E-01	-0.117	9.19E-01	-1.384	5.96E-06	-1.392	4.57E-06
FANCD2	-1.001	4.40E-03	-0.164	9.87E-01	-0.319	8.01E-01	-1.723	1.13E-05	-1.718	9.97E-06
FANCG	-1.131	5.38E-03	-0.150	9.90E-01	-0.264	8.73E-01	-0.760	3.95E-02	-0.915	1.35E-02
FEN1	-1.117	1.96E-04	-0.422	8.72E-01	-0.492	4.82E-01	-0.952	4.27E-04	-1.039	1.55E-04
GADD45A	-0.141	8.57E-01	0.892	9.07E-01	1.112	4.65E-01	0.575	3.15E-01	0.655	2.48E-01
GADD45G	-1.025	2.45E-01	0.002	1.00E+00	0.187	9.58E-01	0.649	3.77E-01	0.449	5.68E-01
H2AFX	-1.404	1.81E-03	-0.365	9.73E-01	-0.354	8.27E-01	-0.691	7.82E-02	-0.808	3.91E-02
HUS1	0.349	2.83E-01	0.252	9.79E-01	0.217	8.70E-01	0.150	6.45E-01	0.089	8.00E-01
LIG1	-1.281	3.29E-04	-0.237	9.85E-01	-0.314	7.87E-01	-1.178	3.42E-04	-1.277	1.32E-04

MAPK12	-1.379	6.35E-04	-0.181	9.87E-01	-0.231	8.85E-01	-0.874	1.33E-02	-1.014	4.48E-03
MBD4	-0.144	7.74E-01	-0.106	9.93E-01	-0.030	9.88E-01	-0.663	8.87E-02	-0.589	1.29E-01
MCPH1	-0.088	7.31E-01	0.071	9.93E-01	0.118	9.06E-01	0.043	8.57E-01	0.071	7.61E-01
MDC1	0.059	8.79E-01	0.216	9.87E-01	0.081	9.54E-01	0.279	3.25E-01	0.393	1.54E-01
MLH1	-0.535	1.57E-02	-0.178	9.79E-01	-0.259	7.18E-01	-0.372	6.84E-02	-0.422	3.76E-02
MLH3	0.275	7.08E-01	0.104	9.93E-01	0.203	9.45E-01	-0.668	2.76E-01	-0.599	3.37E-01
MPG	-0.458	4.36E-01	-0.147	9.93E-01	-0.088	9.72E-01	0.684	1.59E-01	0.581	2.40E-01
MRE11A	-0.226	7.15E-01	-0.206	9.90E-01	-0.038	9.88E-01	-1.131	2.62E-02	-1.201	1.80E-02
MSH2	-0.452	5.45E-01	-0.133	9.93E-01	-0.036	9.92E-01	-1.511	2.20E-02	-1.477	2.33E-02
MSH3	-0.145	6.86E-01	-0.043	9.94E-01	-0.081	9.55E-01	-0.071	8.33E-01	-0.094	7.81E-01
NBN	-0.199	8.54E-01	-0.174	9.93E-01	0.040	9.94E-01	-1.610	5.90E-02	-1.633	5.39E-02
NTHL1	-1.079	5.00E-02	-0.408	9.84E-01	-0.446	8.36E-01	-0.116	8.44E-01	-0.391	4.64E-01
OGG1	-0.797	7.41E-02	-0.066	9.94E-01	-0.206	9.16E-01	-0.521	2.12E-01	-0.359	4.06E-01
PARP1	-0.779	3.29E-03	-0.126	9.87E-01	-0.261	7.75E-01	-0.491	4.04E-02	-0.504	3.40E-02
PCNA	-1.158	6.86E-05	-0.483	7.46E-01	-0.511	3.95E-01	-1.355	4.21E-06	-1.461	1.45E-06
PMS1	-0.314	5.92E-01	-0.223	9.87E-01	0.101	9.67E-01	-1.526	3.94E-03	-1.448	5.18E-03
PMS2	-0.001	9.98E-01	0.117	9.87E-01	0.024	9.80E-01	0.037	8.66E-01	0.126	5.24E-01
PNKP	-1.703	4.46E-03	-0.217	9.90E-01	-0.167	9.49E-01	-1.128	3.48E-02	-1.222	2.22E-02
PPM1D	-0.417	2.02E-01	-0.002	9.99E-01	0.050	9.72E-01	-0.488	1.02E-01	-0.347	2.53E-01
PPP1R15A	0.228	7.87E-01	1.374	6.70E-01	1.555	2.79E-01	1.870	3.24E-03	1.996	1.65E-03
PRKDC	-0.068	9.34E-01	0.306	9.87E-01	0.192	9.47E-01	-0.660	2.74E-01	-0.499	4.23E-01
RAD1	-0.513	1.04E-02	-0.092	9.87E-01	-0.162	8.39E-01	-0.598	2.07E-03	-0.575	2.57E-03
RAD17	0.312	6.86E-01	-0.056	9.95E-01	0.193	9.52E-01	-0.804	2.19E-01	-0.810	2.16E-01
RAD18	-0.300	6.68E-01	-0.128	9.93E-01	0.000	1.00E+00	-1.054	6.91E-02	-1.244	3.28E-02
RAD21	0.034	9.66E-01	-0.096	9.93E-01	-0.034	9.91E-01	-0.885	1.10E-01	-0.806	1.45E-01
RAD50	0.919	2.54E-01	-0.014	9.99E-01	0.527	8.75E-01	-0.146	8.73E-01	0.036	9.71E-01
RAD51	-1.300	1.32E-03	-0.268	9.87E-01	-0.482	6.96E-01	-0.971	7.91E-03	-1.058	3.90E-03
RAD51B	-0.537	1.27E-01	-0.034	9.95E-01	-0.364	7.62E-01	-0.439	1.77E-01	-0.272	4.24E-01
RAD9A	-0.217	5.32E-01	-0.061	9.93E-01	-0.123	9.32E-01	-0.081	8.12E-01	-0.069	8.44E-01
RBBP8	0.015	9.90E-01	-0.430	9.87E-01	-0.159	9.67E-01	-1.495	6.21E-02	-1.609	4.42E-02
REV1	0.050	9.06E-01	0.006	9.99E-01	0.023	9.89E-01	-0.460	1.38E-01	-0.471	1.28E-01
RNF168	-0.149	7.81E-01	-0.110	9.93E-01	-0.117	9.55E-01	-0.675	1.05E-01	-0.678	1.01E-01
RNF8	-0.096	7.22E-01	-0.141	9.87E-01	-0.138	8.92E-01	0.263	2.13E-01	0.215	3.21E-01
RPA1	-0.535	1.46E-02	-0.220	9.59E-01	-0.367	5.34E-01	-0.315	1.19E-01	-0.344	8.56E-02
SIRT1	0.075	9.25E-01	0.192	9.93E-01	0.276	9.19E-01	-0.480	4.38E-01	-0.464	4.61E-01
SMC1A	-0.392	2.68E-01	0.107	9.93E-01	-0.001	1.00E+00	-0.612	5.60E-02	-0.553	8.10E-02
SUMO1	-0.146	7.77E-01	-0.327	9.83E-01	-0.208	9.15E-01	-0.910	2.48E-02	-0.946	1.89E-02
TOPBP1	0.063	9.53E-01	-0.007	9.99E-01	0.070	9.85E-01	-1.270	9.66E-02	-1.324	8.12E-02
TP53	0.386	2.87E-01	0.172	9.87E-01	-0.004	9.99E-01	0.487	1.33E-01	0.261	4.53E-01
TP53BP1	0.343	3.12E-01	0.374	9.52E-01	0.330	7.76E-01	0.373	2.21E-01	0.484	1.05E-01
TP73	-0.891	2.45E-01	-1.152	9.03E-01	-1.080	6.38E-01	-0.924	1.86E-01	-1.293	6.51E-02

UNG	-0.505	6.59E-02	-0.175	9.87E-01	-0.371	6.73E-01	-0.282	2.77E-01	-0.334	1.96E-01
XPA	-0.163	7.27E-01	-0.209	9.87E-01	-0.106	9.54E-01	-0.791	3.63E-02	-1.063	5.98E-03
XPC	-0.455	1.29E-01	0.068	9.93E-01	0.036	9.79E-01	-0.317	2.58E-01	-0.255	3.75E-01
XRCC1	-0.490	2.33E-01	-0.200	9.87E-01	-0.278	8.67E-01	-0.006	9.89E-01	-0.117	7.88E-01
XRCC2	-0.765	2.98E-01	-0.022	9.98E-01	-0.017	9.98E-01	-1.878	8.37E-03	-2.001	5.12E-03
XRCC3	-1.023	5.12E-03	-0.231	9.87E-01	-0.225	8.83E-01	-0.966	5.14E-03	-1.089	1.79E-03
XRCC6	-0.614	7.43E-03	-0.298	9.40E-01	-0.435	4.50E-01	-0.170	4.40E-01	-0.201	3.58E-01
ACTB	-0.295	4.58E-01	-0.027	9.95E-01	-0.137	9.36E-01	0.491	1.38E-01	0.441	1.85E-01
B2M	-0.339	3.46E-01	-0.340	9.59E-01	-0.381	7.45E-01	-0.075	8.41E-01	-0.075	8.44E-01
GAPDH	-0.560	1.24E-01	-0.158	9.87E-01	-0.319	8.15E-01	0.341	3.04E-01	0.276	4.21E-01
HPRT1	-0.385	2.36E-01	-0.293	9.59E-01	-0.352	7.45E-01	-0.509	8.40E-02	-0.496	8.97E-02
RPLP0	-0.324	5.17E-01	-0.302	9.87E-01	-0.451	7.85E-01	0.653	1.06E-01	0.552	1.76E-01

Supplementary Table 4: DNA damage signaling RT-qPCR array Ct values

Target	Cq (DMSO)	Cq (200 nM EPZ)	Cq (1.95 nM Prex)	Cq (2.34 nM Prex)	Cq (1.95 nM Combo)	Cq (2.34 nM Combo)
ABL1	27.92	28.47	28.38	28.18	29.34	29.59
APEX1	23.79	24.58	24.40	24.13	25.16	25.52
ATM	30.87	31.40	32.97	30.62	34.88	35.35
ATR	27.53	28.22	29.09	27.86	31.89	31.89
ATRIP	29.18	30.03	29.46	29.44	31.05	31.16
ATRX	27.70	27.86	29.32	28.01	30.78	30.96
BARD1	27.22	28.81	28.22	27.34	31.44	31.75
BAX	25.67	26.49	26.37	26.21	27.29	27.40
BBC3	32.07	32.38	31.22	31.31	32.71	32.43
BLM	27.17	28.62	28.62	28.01	30.53	31.24
BRCA1	27.99	29.78	29.20	28.36	32.27	32.34
BRIP1	27.93	29.13	29.49	27.95	33.39	33.21
CDC25A	28.31	30.14	29.19	28.91	31.30	31.41
CDC25C	28.37	30.03	28.97	28.85	31.52	31.80
CDK7	26.00	26.18	26.48	25.66	27.91	28.19
CDKN1A	24.46	23.90	24.46	24.16	23.94	24.21
CHEK1	27.98	28.78	29.10	28.17	31.31	31.24
CHEK2	27.93	29.12	28.40	28.28	30.22	30.47
CIB1	24.53	25.22	24.59	24.50	25.33	25.69
CRY1	27.09	27.71	27.87	26.78	29.09	29.35
CSNK2A2	28.00	28.69	27.95	27.64	29.02	29.12
DDB1	25.11	26.03	25.58	25.72	26.15	26.44
DDB2	28.39	29.28	28.17	28.38	29.92	30.10
DDIT3	26.85	26.92	25.89	25.37	26.59	27.09
ERCC1	26.99	28.05	27.13	27.10	27.71	27.95
ERCC2	27.35	28.88	27.55	27.70	29.17	29.46
EXO1	26.87	28.48	27.90	27.17	30.27	30.88
FANCA	28.16	29.23	28.88	28.41	30.32	30.96
FANCD2	27.52	29.40	28.48	28.18	31.34	31.58
FANCG	27.42	29.16	27.94	28.01	30.00	30.50
FEN1	24.41	25.78	25.20	24.83	26.87	27.15
GADD45A	28.26	29.17	27.54	27.08	29.51	29.75

GADD45G	32.17	32.29	32.91	32.98	33.16	32.58
H2AFX	23.74	25.69	24.27	24.38	26.10	26.16
HUS1	28.95	29.59	30.17	29.20	31.82	32.20
LIG1	27.70	29.90	28.22	28.30	30.81	31.03
MAPK12	28.14	30.07	28.73	28.73	30.16	30.96
MBD4	25.68	26.36	26.86	25.95	28.49	28.81
MCPH1	28.20	29.09	29.16	28.55	30.90	30.97
MDC1	28.00	28.57	28.44	28.00	29.75	30.05
MLH1	26.76	28.06	27.47	27.06	29.09	29.41
MLH3	28.93	29.24	30.03	29.07	31.28	31.45
MPG	26.67	27.55	26.64	26.61	27.34	27.78
MRE11A	26.58	27.91	28.22	26.89	30.50	30.68
MSH2	25.76	27.01	27.26	26.09	30.04	30.33
MSH3	27.44	28.25	28.38	27.97	29.64	30.14
NBN	27.09	28.56	28.64	27.37	31.47	31.39
NTHL1	27.34	28.54	27.81	27.84	28.96	29.31
OGG1	29.00	30.91	29.38	29.48	31.61	31.53
PARP1	25.02	26.50	25.82	25.60	27.42	27.76
PCNA	23.25	24.57	24.17	23.67	26.62	27.02
PMS1	26.70	27.58	28.24	26.71	31.16	31.07
PMS2	26.61	27.24	27.41	26.95	28.33	28.36
PNKP	26.57	28.50	26.97	27.02	29.38	29.78
PPM1D	28.44	29.42	29.19	28.58	31.02	31.07
PPP1R15A	26.85	27.20	26.07	25.65	26.59	26.65
PRKDC	25.61	26.84	26.31	25.95	28.91	29.38
RAD1	25.93	26.70	26.30	26.05	28.59	28.88
RAD17	27.90	28.11	29.11	27.61	31.88	32.22
RAD18	27.46	28.15	28.51	27.89	29.70	30.10
RAD21	25.22	26.01	26.54	25.36	28.42	28.72
RAD50	27.42	27.44	28.63	27.30	30.52	30.68
RAD51	27.44	29.02	28.08	27.79	30.13	30.36
RAD51B	28.00	28.44	28.51	28.26	30.02	30.38
RAD9A	29.18	30.06	29.84	29.92	30.91	31.31
RBBP8	26.04	26.48	27.49	26.22	29.80	29.63
REV1	27.39	28.15	28.51	27.49	30.89	31.41
RNF168	27.29	28.23	28.37	27.73	30.26	30.93
RNF8	27.22	27.59	27.98	27.69	28.60	28.93
RPA1	26.11	27.23	26.86	26.51	28.16	28.64
SIRT1	27.56	28.23	28.64	27.87	30.77	31.20
SMC1A	26.11	27.49	27.19	26.74	29.07	29.18
SUMO1	25.08	25.63	26.24	25.33	28.06	28.21
TOPBP1	26.55	27.50	28.17	26.96	30.00	30.46
TP53	29.76	29.75	29.85	29.70	30.59	30.91
TP53BP1	25.82	26.18	26.60	25.91	27.97	28.33
TP73	32.52	33.03	33.75	33.65	34.52	35.04
UNG	25.74	26.20	26.02	25.76	27.31	27.64
XPA	30.21	31.04	31.30	30.07	33.53	33.30
XPC	34.23	34.78	34.28	33.99	35.80	36.78
XRCC1	27.04	27.77	27.52	27.32	28.72	29.04
XRCC2	28.30	29.80	29.55	29.08	32.37	32.50
XRCC3	29.22	30.25	30.06	30.00	31.99	32.42
XRCC6	24.49	25.57	25.47	24.99	26.40	26.68
ACTB	20.30	21.32	21.00	20.97	21.55	21.79
B2M	21.39	22.03	21.90	21.43	23.26	23.59
GAPDH	19.91	21.08	20.63	20.29	21.51	21.74
HPRT1	26.14	27.02	26.89	26.35	28.54	28.92
RPLP0	19.16	19.42	19.36	19.27	19.62	19.88

HGDC	39.00	37.01	38.79	38.56	39.17	Not Detected
RTC	23.32	23.30	23.45	23.73	23.51	23.62
RTC	23.40	23.27	23.52	23.65	23.55	23.65
RTC	23.34	23.22	23.41	23.58	23.54	23.52
PPC	20.05	20.02	20.06	20.09	20.21	20.11
PPC	20.00	20.00	20.01	19.90	19.97	19.95
PPC	19.97	19.98	19.93	19.96	19.89	19.95

Supplementary Figure 1

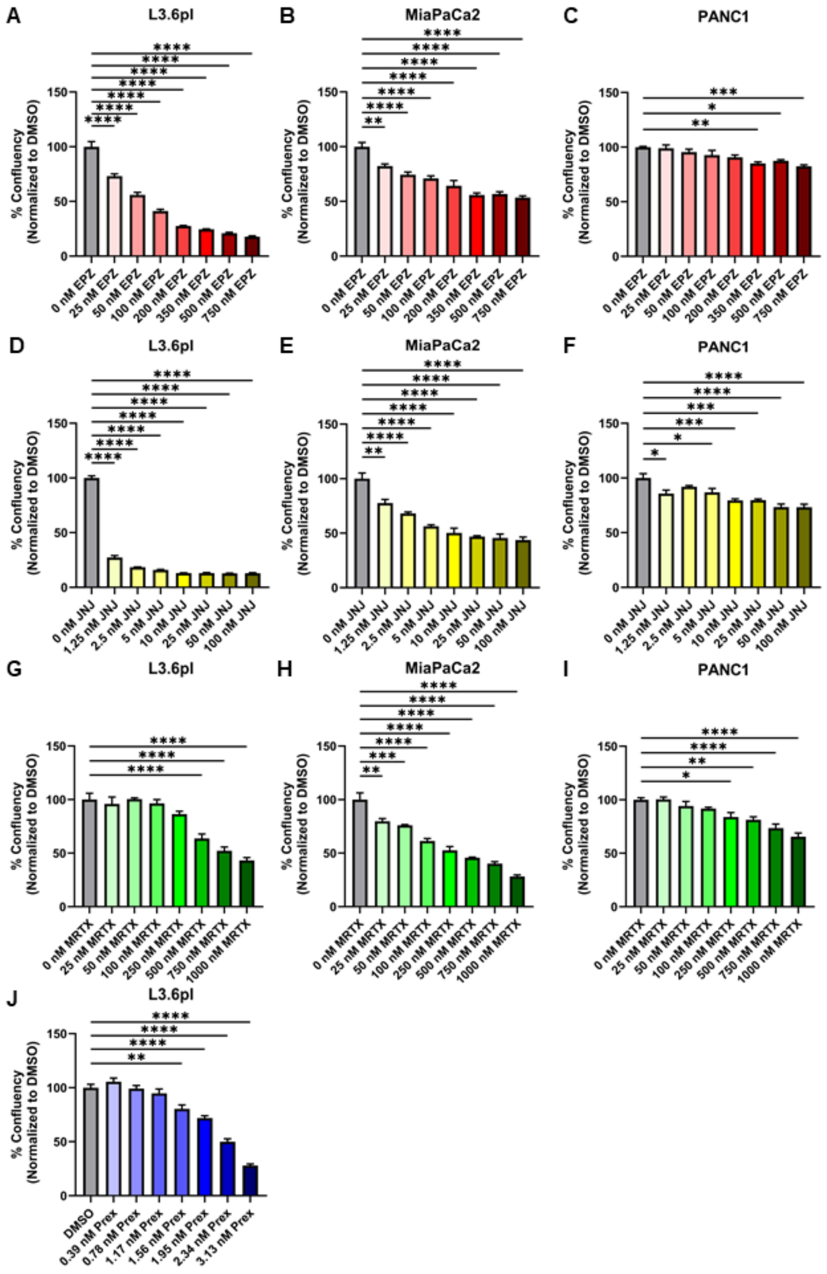

Figure S1: Dose-response analysis of PRMT5 inhibitors across PDAC models defines effective concentration ranges. (A-I) PDAC cell lines L3.6pl, MiaPaCa2, and PANC1 were treated with increasing concentrations of PRMT5 (EPZ015938, JNJ64619178, and MRTX9768) inhibitors. Cell viability was evaluated after 120 hours of treatment via IncuCyte SX5 Live Cell Analysis, and statistical significance was calculated between drug treatment and vehicle (DMSO) control. Mean ± SEM is shown. (n=3; *p≤0.05, **p ≤0.01, ***p≤0.005, and ****p≤0.0001; one-way ANOVA with Tukey's post-hoc). **(J)** L3.6pl cells were treated with increasing concentrations of the CHK1 inhibitor prexasertib to establish working dose range by evaluation of cell viability as described for PRMT5 inhibitors.

Supplementary Figure 2

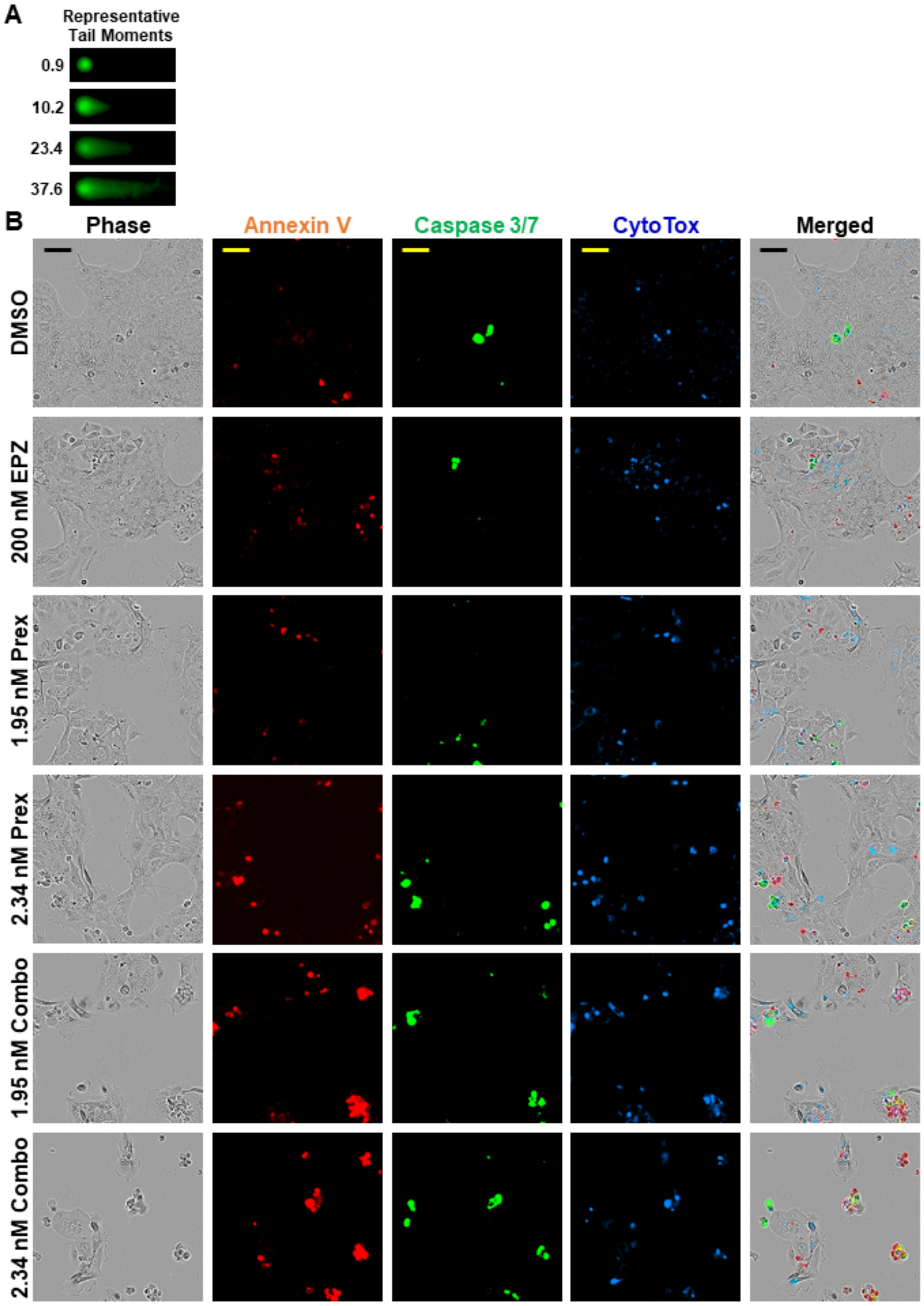

Figure S2: Combined PRMT5 and CHK1 inhibition promotes DNA damage as well as apoptotic and overall cell death in PDAC cells. L3.6pl cells were treated with 200 nM EPZ015938 and 1.95-2.34 nM prexasertib, alone or in combination. **(A)** Neutral comet assay detecting DSBs in L3.6pl cells after 48-hour treatment. Representative comets illustrate the range of tail moments analyzed. **(B)** Cells were incubated with Caspase 3/7 green, Annexin V red, and CytoTox NIR dyes for 96 hours using the IncuCyte SX5 Live Cell Imaging system. Fluorescence for each dye was quantified over time. Representative images from the 96-hour timepoint are shown for brightfield, Caspase 3/7, Annexin V, and CytoTox NIR fluorescence channels (10X magnification; scale = 200 μm).

Supplementary Figure 3

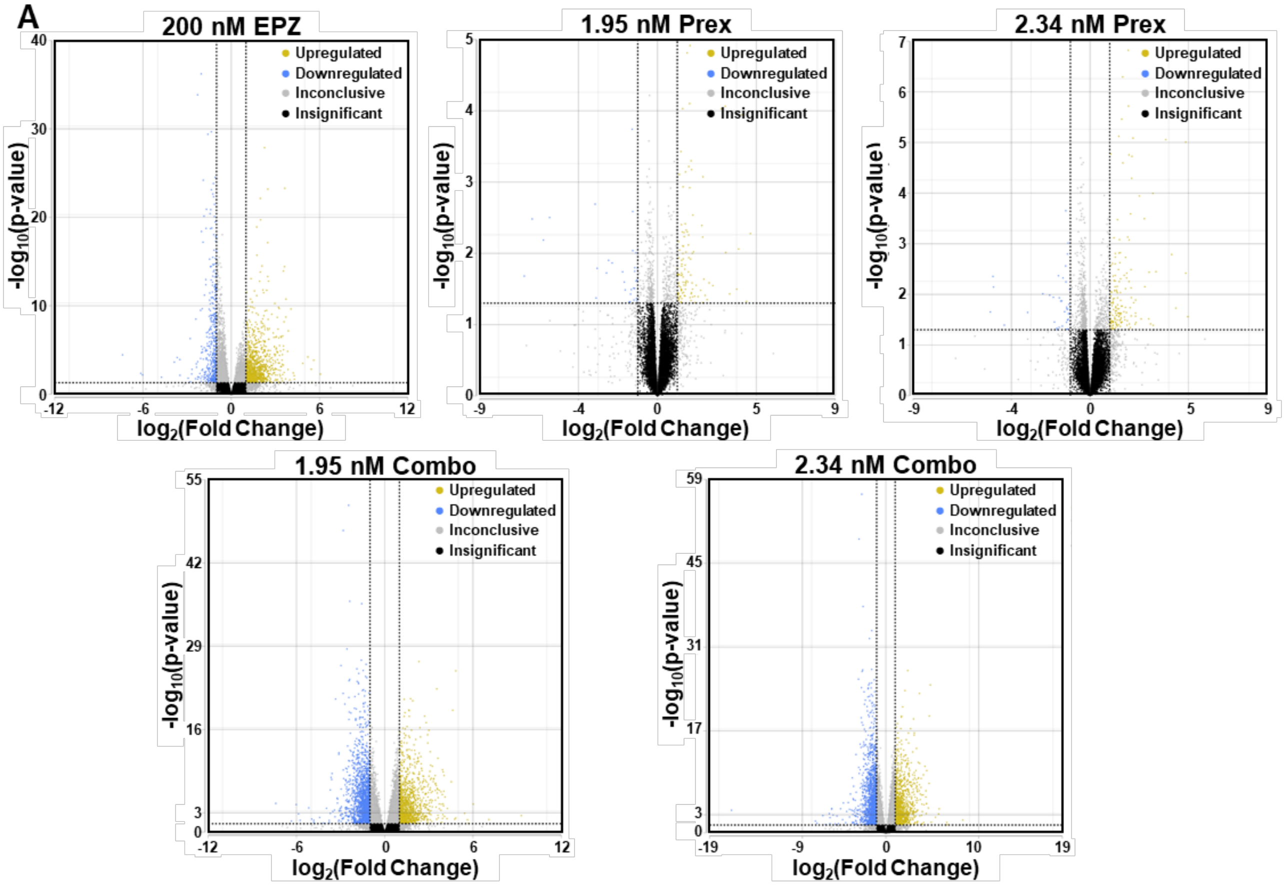

Figure S3: Volcano plots demonstrating trends in upregulated and downregulated genes from RNA-seq analysis. Each dot represents a single gene, where yellow dots indicate upregulated genes, blue dots indicate downregulated genes, grey dots indicate inconclusive changes to regulation, and black dots indicate nonsignificant DEGs (n=3).

Supplementary Figure 4

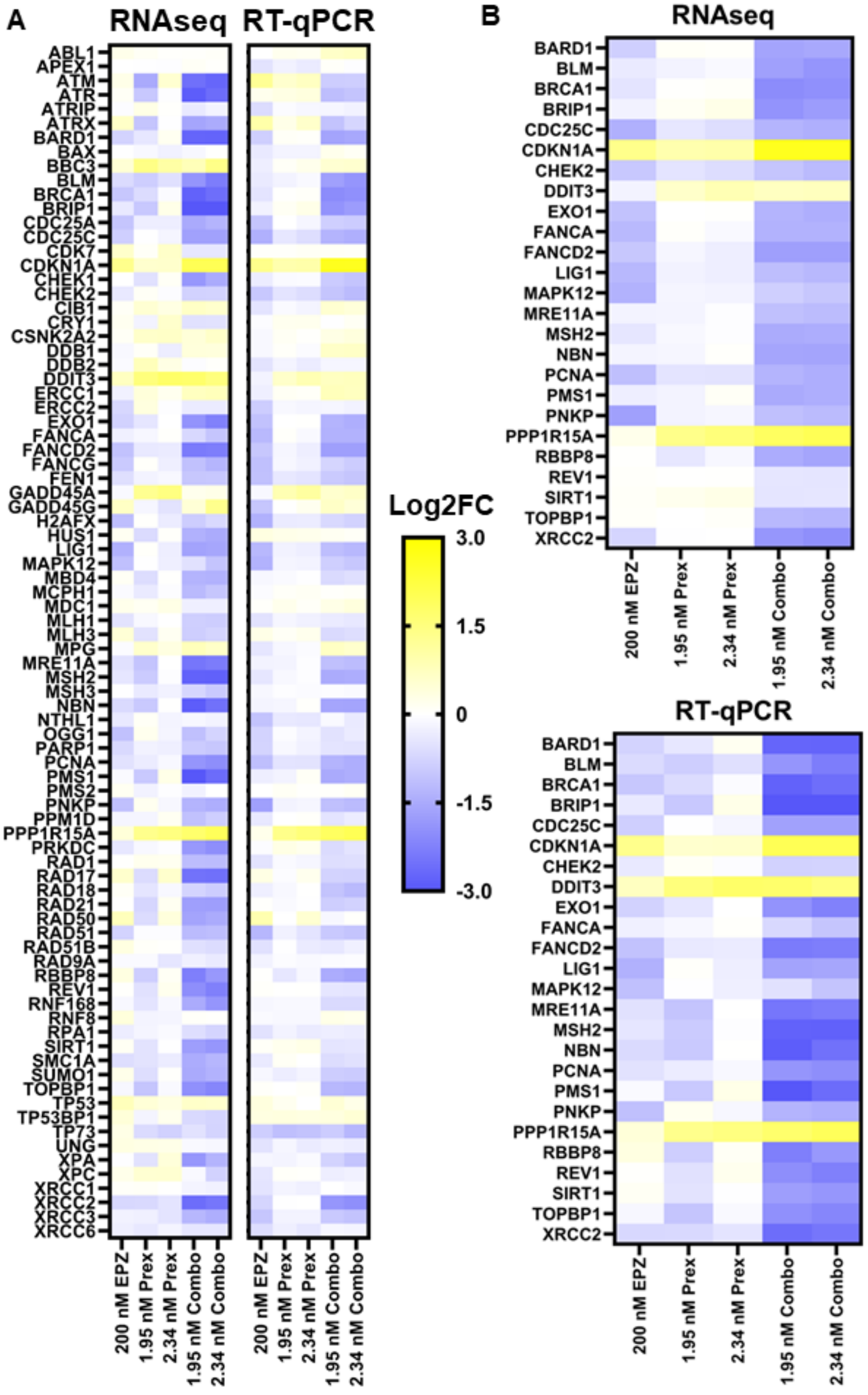

Figure S4: RT-qPCR array validation supports alterations in DNA damage signaling pathways defined by RNA-seq. (A) Heatmap comparing Log2FC values obtained from the RT2 Profiler DNA damage signaling pathway PCR array to those from RNA-seq analysis (n=3). **(B)** Heatmap showing the top 25 DEGs detected by both RNA-seq and RT-qPCR array analyses (n=3).
